# Inositol Hexakisphosphate Promotes Open Pore State in HIV Capsid Protein Lattice by Reconfiguring Subunit Contacts

**DOI:** 10.1021/acsnano.6c07706

**Published:** 2026-09-19

**Authors:** Yari Katar Knelissen, Judith Escrig, Amanda C. Macke, Sruthi Sudhakar, Santos Domínguez-Zotes, Sourav Maity, Mauricio G. Mateu, Juan R. Perilla, Wouter H. Roos, Alejandro Valbuena

**Affiliations:** † Moleculaire Biofysica, Zernike Instituut, Rijksuniversiteit Groningen, 9747 AG Groningen, The Netherlands; ‡ Centro de Biología Molecular Severo Ochoa (CSIC-UAM), Campus of the Universidad Autónoma de Madrid, 28049 Madrid, Spain; § Department of Chemistry and Biochemistry, 5972 University of Delaware, Newark, Delaware 19716, United States; ∥ Departamento de Biología Molecular, 16722 Universidad Autónoma de Madrid, 28049 Madrid, Spain

**Keywords:** high-speed AFM, human immunodeficiency virus, capsid protein lattice, pore opening, molecular dynamics, constant-pH molecular dynamics

## Abstract

The mature human immunodeficiency virus (HIV-1) capsid is composed of approximately 250 capsid protein hexamers, each containing a central pore delimited by a β-hairpin and an α-helix from each monomer. The cellular metabolite inositol hexakisphosphate (IP6) binds this pore and modulates translocation of nucleoside triphosphates into the capsid, where they fuel reverse transcription of the viral genome. However, the mechanism by which IP6 modulates the pore conformation is poorly understood. Averaged structural models of the HIV-1 capsid protein lattice have shed some light on this mechanism but lack information on the dynamic behavior of individual hexamers that could provide deeper mechanistic insights. Here we visualized in real time by high-speed atomic force microscopy the asynchronous motions of individual hexamer pores in the presence or absence of IP6, identifying dynamic transitions between two major states. Quantitative analysis enabled estimation of the IP6-induced pore conformations and populations. Constant-pH molecular dynamics simulations further reveal that IP6 induces contact rewiring at the pore entrance, stabilizing the open state through coordinated rearrangement of intersubunit interactions. Consistently, mechanical fatigue assays show that IP6 markedly enhances the resistance of the capsid lattice to mechanical fatigue, revealing its role as a mechanically stabilized nanomaterial. Together, these results establish IP6 as a proviral regulator that couples nanoscale pore dynamics, intersubunit contact reconfiguration, and mechanical resilience of the HIV-1 capsid, providing a unified mechanistic framework for its role in viral replication.

## Graphical abstract

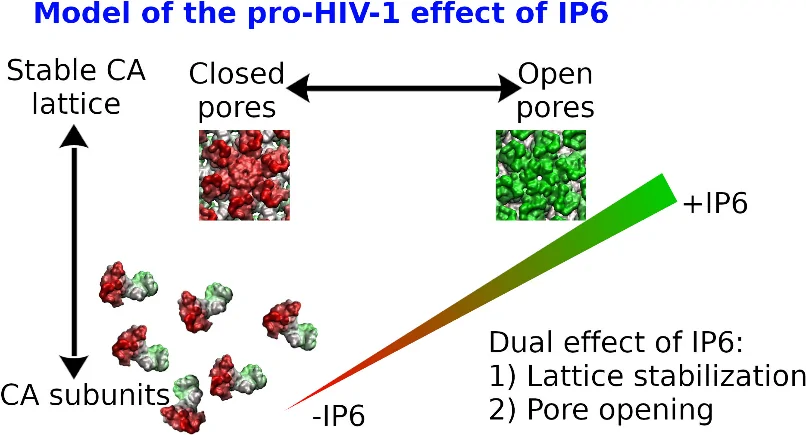

During human immunodeficiency virus type 1 (HIV-1) infection, after a mature virion enters a host cell, the viral core is trafficked to the cell nucleus.^1–3^ During and after this journey, both in the cytosol and in the cell nucleus, viral RNA is reverse transcribed into DNA, an essential step that can be targeted by molecules with antiviral activity.^2,4,5^ The HIV-1 core is made of a pleomorphic cone-shaped capsid composed of approximately 200–250 hexamers and exactly 12 pentamers (Figure 1A) of the capsid protein CA. The CA protein consists of an N-terminal domain (NTD), which contains a β-hairpin, and a C-terminal domain (CTD) connected to the NTD by linker peptide segment (Figure 1B,C). The viral capsid encloses the nucleocapsid complex containing the viral RNA, as well as a number of other viral and cellular proteins, some of which are required for RNA reverse transcription.^6^ A channel or “pore” located at the center of each capsid hexamer (Figure 1B) allows the translocation of ions^7^ and other small molecules, including deoxynucleoside triphosphates (dNTPs) required for DNA synthesis.^8,9^ During HIV-1 virion morphogenesis and maturation, the cellular metabolite inositol hexakisphosphate (IP6) binds to the hexamer pores (Figure 1C), stabilizes the capsid core,^10,11^ and enhances DNA reverse transcription.^9,11,12^

**Figure 1:**
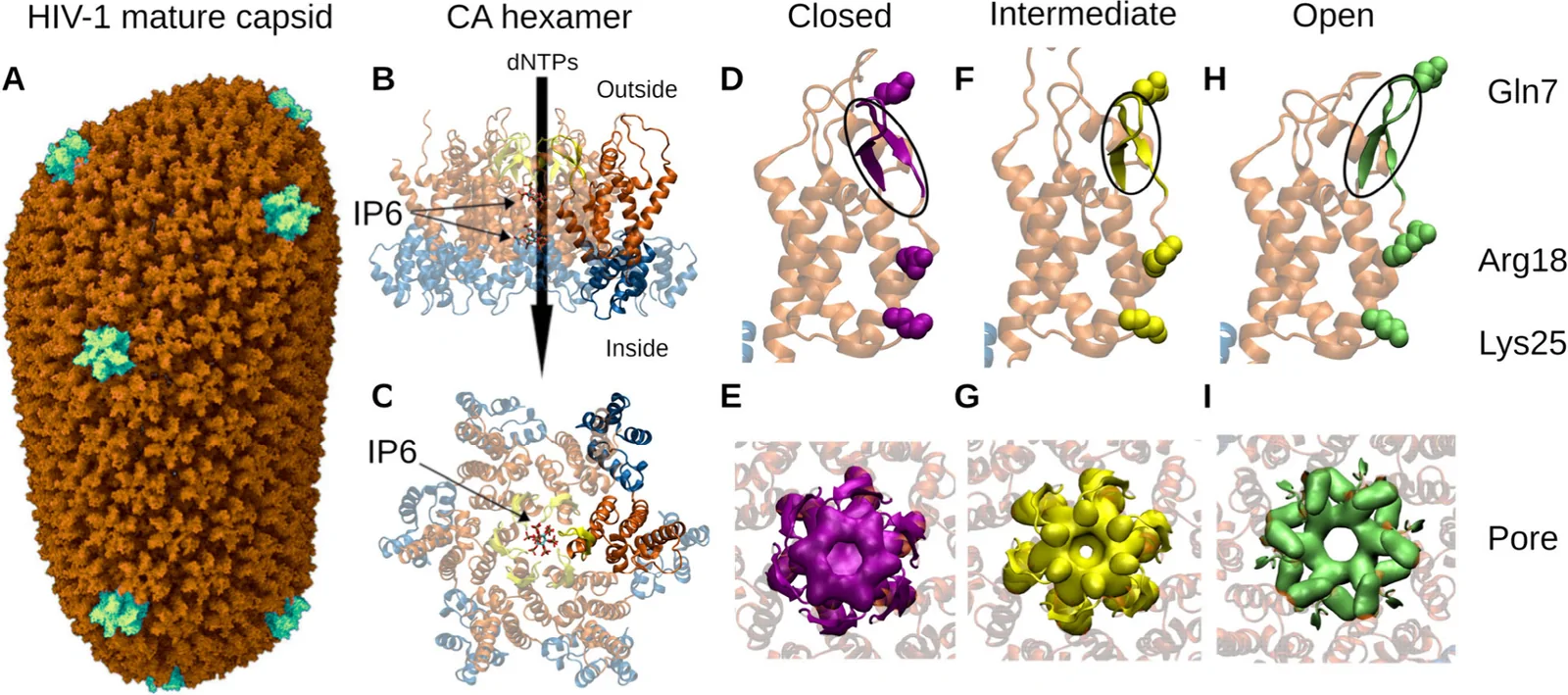
**Structures of the mature HIV-1 capsid, CA hexamer, and region around the pore in the open, intermediate, and closed states.** (A) Cone-shaped HIV-1 capsid composed of ∼200–250 CA hexamers (orange) and 12 CA pentamers (green) (PDB entry 3j3y). (B) Side view of the CA hexamer (one CA subunit is shown in solid color) with the NTD in orange, the CTD in blue, and the β-hairpin in yellow. Two IP6 molecules are shown bound to the hexamer pore. The long thick black arrow shows the most probable pathway dNTP molecules follow from the outside to the inside of the HIV-1 capsid (PDB entry 6bht). (C) Top view of the CA hexamer with two IP6 molecules (only the top one can be seen) bound at the hexamer pore. Colors are as in (B) (PDB entry 6bht). (D–I) Detail of the β-hairpin (ribbon model) enclosed in an oval and the side chains of Gln7, Arg18, and Lys25 (represented as van der Waals (vdW) models) from the side (D, F, H) or from the top (E, G, I) in the closed (purple), intermediate (yellow), or open (green) states of the pore (PDB entries 6b2j, 6b2i, 6es8, and 6bht).

The pore located in the center of the CA hexamer is surrounded by the CA NTD β-hairpins at the top and the residues of helix α1 in the middle and bottom (Arg18 and Lys25 rings, respectively), and these elements delimit the minimum diameter of the pore (Figure 1D,F,H). The analysis of hexamer structures determined under different conditions,^12–15^ either in the presence or in the absence of IP6, indicates that the β-hairpin is statically and/or dynamically disordered and that it may undergo large-amplitude motions^16^ that could alter the permeability of the core (Figure 1D–I),^17^ and influence the mechanisms by which it engages cellular factors in the cytoplasm for its transport to the cellular nucleus.^18,19^ Structural and biophysical studies have shown that the N-terminal β-hairpin exhibits intrinsic conformational flexibility across isolated CA proteins, *in vitro* assemblies, and native capsids,^9,12,13,15^ and this conformational variability is thought to couple transient hexamer “breathing” motions to pore opening and permeability.^20,21^ Recent cryo-EM studies have shown that CA hexamers are intrinsically curved, flexible, and asymmetric, contributing directly to capsid curvature,^22^ and that their conformational properties can vary depending on local curvature within the capsid lattice,^21^ or even the presence of pentamers.^23^ Therefore, while planar assemblies capture the essential intra-hexamer structural features and dynamics, curvature-dependent effects present in native capsids may further modulate pore dynamics.

A crystallized CA protein lattice containing the amino acid substitution E45A was found with pores in either the closed or the open conformation.^13^ Major differences between the E45A CA mutant structures capture different arrangements of the β-hairpin and the diameter of the Arg18 and Lys25-delimited diaphragms inside the pore.^13^ This observation suggested that the β-hairpin conformational state can be taken as an indicator for the pore state (Figure 1D–I). In the hexamer structure solved in the presence of IP6, the pore and the β-hairpin are found in an intermediate state (Figure 1F,G),^12,24^ suggesting that IP6 binding to the CA hexamer may regulate the β-hairpin conformation and the state of pore opening. It has been proposed that CA lattice stabilization by IP6,^11,12,23,25–27^ cooperative binding of IP6 and dNTPs,^9^ or modulation of the electrostatic potential^28^ may enhance nucleotide fueling inside the capsid and, thus, reverse transcription of the viral genome.^26^

It has been posited that the conformations of the hexamer pores can also be controlled through an acidity sensor located close to the entry of the pore.^13,15^ The closed state is promoted at pH > 7. Under this condition, it has been observed that CA residues H12, T48, D51 and Q50 form a tetrahedral hydrogen bond network that involves a water molecule, the β-hairpin is in a position compatible with a closed state. In contrast, at pH < 7 the open state is favored. In the latter, the water molecule in the tetrahedral network is displaced, permitting a direct interaction between H12 and D51, changing the angle of the β-hairpin and modulating the pore constriction.^15^ Although the HIV-1 core is not expected to experience a pH drop during cytosolic transport,^29,30^ due to the proximity of the IP6 binding sites to the pH sensor in the CA hexamer, a study of the interaction between IP6 and the pH sensor at the pore entrance could provide insights into the mechanisms by which the β-hairpin conformation and pore opening can be modulated in the presence of the physiologically relevant metabolite IP6.

The above results were obtained using mainly averaging structural techniques, which limited the elucidation of the conformational dynamics of the β-hairpin as a function of the pH or the presence of IP6. In this study, using high-speed atomic force microscopy (HS-AFM), we have visualized the conformational dynamics of the mature HIV-1 CA protein network in real time at high resolution and have carried out single-particle analysis (SPA) to quantify the proportion of β-hairpin conformations compatible with open or closed pores, under basic or acidic conditions and in the absence or presence of IP6. This powerful approach can be applied to other biomolecular systems; in the case of the results presented here for the HIV capsid protein lattice, the findings show that both pH and the binding of IP6 to the CA pores promote pore opening. To understand the pore dynamics and its conformational state after acidification and IP6 binding in atomic detail, all-atom constant-pH molecular dynamics (CpHMD) simulations were performed in the absence or presence of IP6. The results revealed a strong dependency on pH and influence of IP6 on the H12_*i*_–E45_*i*+1_–D51_*i*_ triad involved in CA–CA binding. Finally, mechanical analysis of the CA lattice by AFM revealed that IP6 binding to the CA protein lattice limits its conformational flexibility and dramatically increases its resistance to material fatigue. Taken together, the results of this study support a double role for IP6 as a proviral metabolite which: (i) promotes the opening of the CA pores by modulating the population of the open and closed states by rewiring the CA–CA contact network, and (ii) increases the resistance of the CA protein lattice against disruption by material fatigue.

## Results and Discussion

### Imaging of the CA Protein Lattice by HS-AFM Reveals That the Pore Region in Each Hexamer Is Highly Dynamic

The flat, single molecule thick, hexagonal CA protein lattice that forms the mature HIV-1 capsid was self-assembled from recombinantly expressed and purified CA on an atomically flat, freshly cleaved mica substrate.^31^ The assembly reaction was carried out in phosphate buffer (50 mM, pH 8) supplemented with 137 mM NaCl, using a low total CA concentration (1 μM) and a long incubation time (1 h) to minimize the occurrence of defects in the protein lattice. These conditions favored the self-assembly of CA patches sufficiently large and defect-free, suitable for imaging (Figure 2A). Then several lattice patches were imaged using HS-AFM (see Methods).^32^ Lattice gaps were used to measure the height of the CA lattice. The value obtained (*h* = 6.3 ± 0.9 nm, *N* = 24) was virtually the same as that obtained previously by AFM^31^ and other structural techniques.^14,33^ The average distance between contiguous hexamers (*b* = 9.5 ± 0.3 nm, *N* = 264) was used for image calibration (so that the hexagonal lattice parameter was consistent with that previously determined), which allowed the identification of subtle structural changes from AFM images.

**Figure 2:**
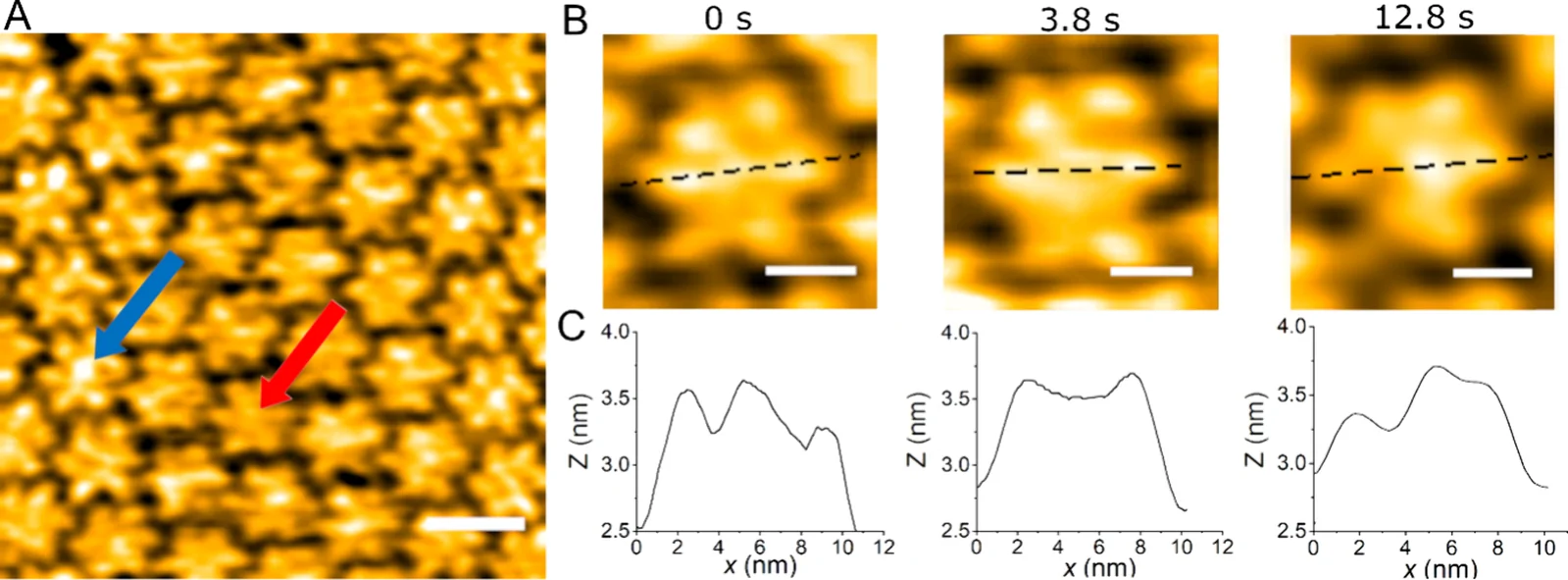
**Conformational dynamism of CA hexamers visualized by HS-AFM at pH 8.** (A) Single HS-AFM frame showing hexamers with a bulge (blue arrow) or a flat region (red arrow) at their center. The scale bar corresponds to 10 nm. (B) Time evolution of a single hexamer, revealing its transition from state 1 (bulge present, left) to state 2 (no bulge, middle) and from state 2 to state 1 again (right). Scale bars correspond to 3 nm. Acquisition times are indicated for each frame. (C) Height profiles corresponding to the dashed lines traced over the hexamers in (B), which show small but quantifiable height differences between the hexamer’s center to Cyp-A loop (0.5 nm, left; 0.2 nm center; 0.5 nm right).

Visualization by HS-AFM of the CA protein lattice in real time showed a rather complex dynamic behavior (Movie S1). Single star-shaped hexamers showed uncorrelated motion of the six lobes that changed their morphology, including their height at the sixfold symmetry axis where the pore is located (Movie S2). Considering that the functional state of the pore is linked to the structural state of the β-hairpins which surround the pore at the center of each hexamer^15^ (see above and Figure 1D–I), special attention was devoted to the analysis of this central region. A seventh lobe in the center of the hexamer, hereafter referred to as the bulge, was transiently observed. Continuous imaging of a same hexamer showed that the center of the hexamer fluctuated between two states, one in which the bulge was observed, and another one in which the bulge was not detected, and the central region was nearly flat (Figure 2B). Given the small tip size (∼2 nm), convolution effects are expected to have only a minimal impact on the apparent lateral dimensions of the bulge. Topographic changes at the hexamer center were <0.5 nm in height (Figure 2C), of the same order of the changes previously observed in the β-hairpin conformation (see Introduction) and remain reliably detectable given the vertical resolution of HS-AFM under our imaging conditions. The time involved in the transition between the conformational states observed by HS-AFM (state 1, central bulge; state 2, no bulge, flat center) in this example was *t* ≈ 4–8 s (Figure 2B). This time was clearly longer than the acquisition time for each frame *t*_frame_ = 0.9–1.3 s, which yielded enough temporal resolution to analyze the observed conformational changes as a function of time under the experimental conditions used. The possibility of transitions faster than *t*_frame_ limits the characterization of transition kinetics. However, the identification and relative populations of the conformational states remain robust, as these are observed as stable configurations persisting over multiple frames. Under our imaging conditions, tip–sample interaction forces are expected to be as low as possible and comparable across experiments and are therefore unlikely to selectively stabilize one conformational state over the other.

In summary, topographic images of the hexameric HIV-1 CA protein lattice obtained with high temporal resolution showed a high degree of dynamism for individual hexamers, which fluctuated between two major conformational states characterized by the presence (state 1) or absence (state 2) of a bulge at the hexamer central region, where the six β-hairpins and the pore are located.

### The Bulge (State 1) and Flat (State 2) Conformational States of the CA Hexamer Are Assigned to the Open (O) and Closed (C) States of the Central Pore, Respectively

To assign the two conformational states of the CA hexamer observed by HS-AFM (the bulge state, state 1, and the flat state, state 2) to the two functional hexamer states (that with an open pore (O) or that with a closed pore (C)), the following approach was undertaken. Hexamer conformation and pore functionality are related^15^ (Figure 1D,I), and population of these states can be promoted by adjusting environmental conditions, such as the pH and the absence or presence of IP6.^12,15^ First, a change in the acidity of the medium from pH 8 to pH 6 was introduced, as these pH values are respectively below and above the proposed pH threshold for β-hairpin tilt and pore opening.^15^ SPA was performed to automatically identify and classify the number of hexamers in state 1 or in state 2. Because of the high vertical (*Z*) resolution of HS-AFM images, subnanometer resolution features could be detected, and the presence or absence of the bulge in many imaged hexamers (which may depend on the conformational state of the β-hairpins) could be ascertained.

To perform SPA with the CA lattice, each hexamer was considered as an individual particle. First, individual hexamers were selected and classified according to their topography. For this purpose, high-resolution HS-AFM images of the CA lattices were obtained, and the cryo-EM analysis software package Scipion 3.0 ^34^ for SPA was used (Figure 3). When interpreting AFM images with software designed to understand electron transmission images, the *Z*-scale information is lost, and therefore, the averaged images obtained from the classes representing the pore states will be expressed in arbitrary units. However, the *Z* range of all videos and all conditions, corresponding to the top of the CA lattice, was approximately 1.2 nm. High-resolution HS-AFM movies were divided into frames, and images of individual hexamers were extracted. Several rounds of classification were performed using *cl2d* ^35^ with a variable number of classes to remove incomplete and poorly resolved particles (Figures 3 and S1 and Table S1). The final classification step was performed with six classes (Figures S2, S3, and S5), and height profiles were obtained to determine whether that class showed a bulge or a flat region in the center of the hexamer. Finally, the hexamer images belonging to those classes with either a state 1 or state 2 center were averaged.

**Figure 3:**
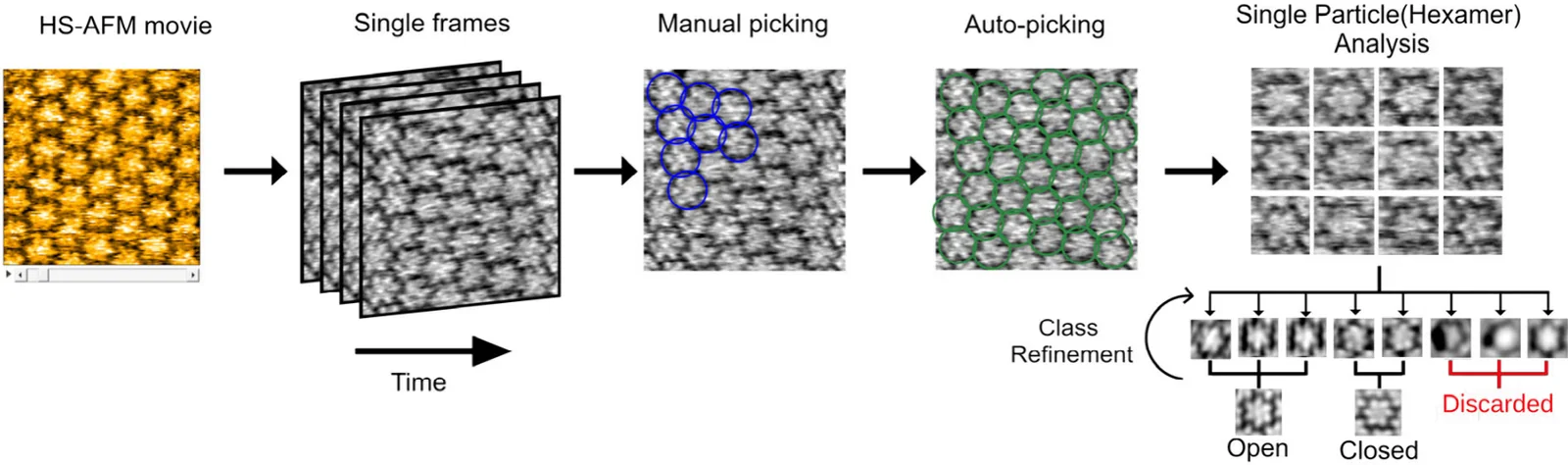
**Workflow for single-particle analysis from HS-AFM movies.** First, an HS-AFM movie was acquired and divided into individual frames. In each frame, a few hexamers (blue circles) were manually picked, followed by automatic particle picking based on deep learning (green circles). Comparison of the topography of individual hexamers was used for classification into several classes to eliminate bad (incomplete and/or insufficiently resolved) particles (indicated in red). Class averaged HS-AFM images of open and closed pores were obtained by averaging those classes with open or closed pores.

Experiments performed at pH 8 in the absence of IP6 showed that 64 ± 7% of the hexamers displayed a flat central region (state 2) and 36 ± 7% exhibited a central bulge (state 1). To illustrate the classification procedure, the average images and height profiles of the six final classes obtained at pH 8 are shown in Figure S2. In the classification performed by user 1, classes I, II, and V were assigned to state 2, whereas classes III, IV, and VI corresponded to state 1. Comparable results were obtained independently by user 2, and the exact population values are reported in Table S1.

The above results showed that at pH 8 in the absence of IP6, states 1 and 2 differ substantially in population, yet the overlap between them prevents an unambiguous assignment of conformational (1/2) to functional (O/C) states. Thus, a new series of experiments were performed at pH 6 to promote pore opening.^15^ SPA at pH 6 showed a drastic change in the proportion of hexamers in state 1 or 2. The two independent analyses found most of the hexamers (98.9 ± 0,3 %) in state 1 (Figure 4A, Table 1, and Figure S3). Only a marginal fraction of hexamers (1.1 ± 0.3 %, corresponding to 17 ± 14 particles) could be assigned to state 2 and subsequently removed before calculating the average image (Figure 4A, center).

**Figure 4:**
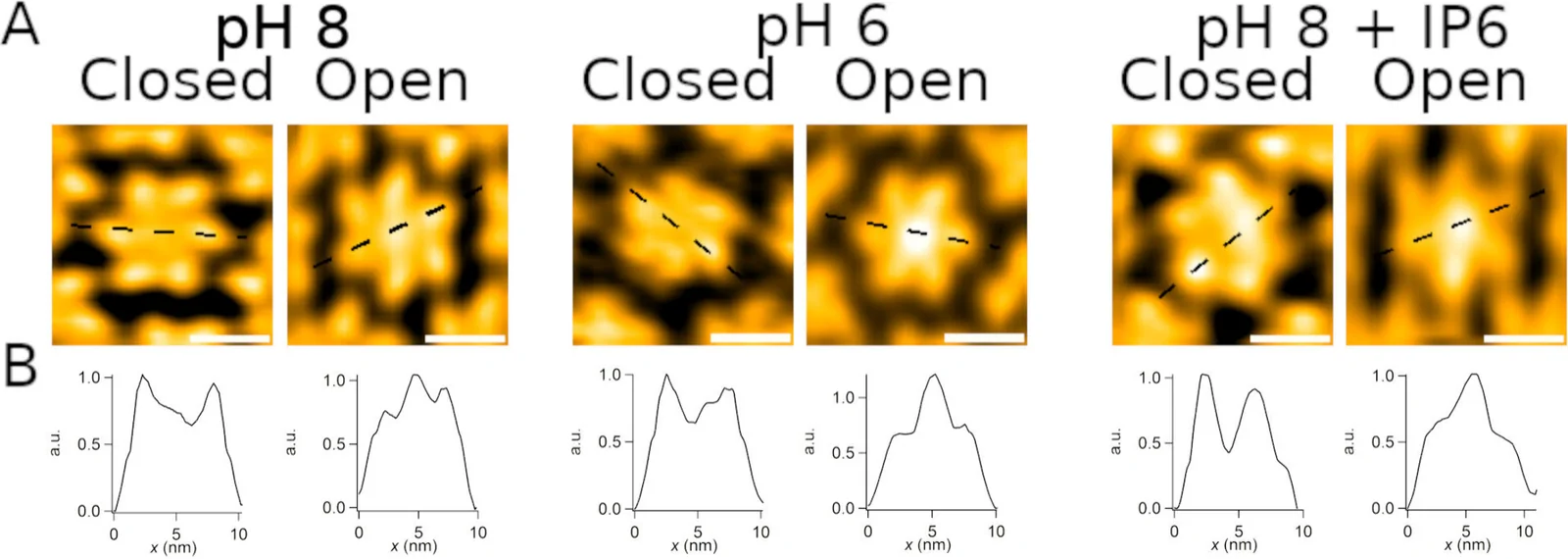
**Hexamers with open or closed pores.** (A) Averaged topography of hexamers in a closed (C) or open (O) state at pH 8, pH 6, or pH 8 + 1 mM IP6. Scale bars correspond to 5 nm. (B) Topographic profiles following black dashed lines in (A) for the averaged hexamers in a C state (which shows a central flat region) or in an O state (which shows a central bulge).

**Table 1:** Number and proportion of hexamers with open or closed pores, obtained from two independent analyses under the conditions tested

	closed state			open state		
	*N*_C_ ^a^			*N*_O_ ^c^		
condition	user 1	user 2	%^b^	user 1	user 2	%^b^
pH 8	1422	984	64 ± 7	992	448	36 ± 7
pH6	27	7	1.1 ± 0.3	1965	793	98.9 ± 0.3
pH 8 + IP6	1269	926	20 ± 3	5998	3305	80 ± 3

a *N*_C_ is the number of hexamers with closed pores.

b % indicates the relative abundance of hexamers with open or closed pores under the conditions tested. The % values are reported as average ± standard deviation.

c *N*_O_ is the number of hexamers with open pores.

Since hexamers from the CA E45A mutant have been structurally resolved in distinct conformations showing either a more open or a more closed pore (Figure 1D,H),^13^ we used these structures of the E45A hexamer (PDB entries 6b2j and 6b2i) to simulate AFM-like images (see Methods), plot the hexamer height profiles (Figure S4), and compare the experimental and simulated profiles to assign closed or open pores. It should be noted that while the E45A mutation removes the E45 residue involved in the pH sensor, it also relaxes local electrostatic constraints and enables the pore to sample broader conformational space.^13^ Therefore, these structures are interpreted here as representative configurations of open and closed states rather than direct equivalents of WT pH-dependent states. Average experimental images of CA hexamers with closed or open pores at pH 8 are shown in Figure 4A. By analyzing the height profiles of the hexamers with open or closed pores, it can be observed that the central region of the hexamer is depressed if the pore is closed, but shows a central bulge if the pore is open (Figure 4B). The simulated images and profiles, although not perfectly matching the experimental ones, follow the same trend in which the center of the hexamer is flatter and deeper in the closed state than in the open state (Figure S4). These results indicate that hexamer state 2 (flat center) corresponds to the C state (closed pore), and hexamer state 1 (central bulge) corresponds to the O state (open pore).

In summary, SPA of CA hexamers using HS-AFM led to the identification of two major conformational states. At pH 8, states 1 and 2 were populated to substantially different but still overlapping extents, whereas at pH 6 state 1 was overwhelmingly favored, comprising 98.9% of the hexamers. Since the open pore state predominates in acidic conditions^15^ and the comparison between the simulated and experimental hexamer topographies showed a lower height in the closed pore state than in the open pore state (Figure S4), state 1 is assigned to the open pore state (O), and state 2 to the closed pore state (state C). Hereafter, state 2 will be considered to be equivalent to the closed state C, and state 1 will be considered to be equivalent to the open state O. The assignment of states 1 and 2 to pore conformations potentially compatible or incompatible with dNTP passage is based on HS-AFM topography and should not be interpreted as a direct measurement of pore permeability. Thus, the central protrusion is considered a conformational signature rather than definitive evidence of a functionally open pore. Intermediate states or alternative β-hairpin arrangements may remain unresolved or generate similar topographic signatures.

### Binding of IP6 to the CA Lattice Promotes Pore Opening at Close to Physiological pH

Binding of IP6 to the HIV-1 capsid promotes DNA synthesis within the virion and increases capsid stability.^11,12^ In the present study we evaluate the possibility that IP6 binding to the CA protein lattice could also promote the opening of the hexamer pore. The expected stoichiometric ratio between IP6 and the CA hexamer is 1:1,^12,24^ and under our experimental conditions, IP6 is anticipated to occupy the CA hexamers at nearly saturating levels. HS-AFM imaging of the CA lattice in the presence of IP6 was performed as described above for the CA lattice in the absence of IP6. After completion of the self-assembly reaction, the sample was incubated with 1 mM IP6 at pH 8. HS-AFM imaging revealed a dynamic behavior that was similar to that previously observed at pH 8 in the absence of IP6: (i) different hexamers in the lattice at any instant in time were found in either the C or O states, and (ii) a given hexamer could fluctuate between the C and O states. Thousands of individual hexamers were extracted from the HS-AFM movies and classified into six classes (Figure S5 and Table S1). Topographic profile analysis of the class-averaged images identified five classes that were consistent with the O state of the pore, and only one class was assigned to the C state (Figure S5). The population of hexamers in either the O or C states was quantified, revealing that 80 ± 3% corresponded to the O state and 20 ± 3% to the C state (Figure 4A and Table 1). In summary, in the presence of IP6 at pH 8, the proportion of hexamers in which the pore is open increased compared to their proportion in the absence of IP6 at the same pH (Table 1). The shift in the population of each hexamer state reflects that the free energy difference between open and closed states is modulated by the binding of IP6 (see the Supporting Information).

Given that pH and IP6 similarly modulate β-hairpin conformation, pore opening energy, and pore state distribution, we wondered how the pH sensor, the presence of IP6, and pore opening are related. To determine the possible influence of IP6 and/or titratable side chains in the protonation states, we performed p*K*_a_ calculations with default settings with the H++ server using the CA hexamer complexed with IP6 (PDB entry 6bht). These results showed that residues P1, R18, H12, K25, E45, and D51 are close enough to each other to influence the charge state of the others. It is important to note that residues H12, E45, and D51, which participate in the establishment of the tetrahedral hydrogen bond,^13,15^ are very close to the residues that interact with IP6, R18, and K25. Therefore, the binding of IP6 could critically affect the net charge of these residues and, consequently, the conformation of the β-hairpin. To explore this further, we performed CpHMD simulations which enable explicit sampling of protonation states of the given residues.^36^

### Constant-pH Molecular Dynamics Reveals Critical pH- and IP6-Dependent Behavior for Capsid Pore Dynamics

The central pore channel, spanning from the exterior to the interior of the CA protein capsid, is formed by residues of the six β-hairpins together with key residues belonging to αH1, including R18 and K25, which are known to bind and regulate IP6. Structural analyses have revealed a finely tuned interaction network at the base of these hairpins, including a conserved P1–D51 salt bridge^37,38^ and surrounding hydrophilic interaction contacts involving P1, H12, A14, and T48.^39,40^

Contributions from the neighboring protomer, particularly between E45_*i*+1_ and H12, Q50, and D51,^13^ further stabilize this region. Interestingly, it has been suggested that dynamic pore dilation may be regulated in a pH-dependent manner through a water-mediated hydrogen-bonding network linking H12, T48, Q50, and D51.^14^ Among these residues, H12, E45, and D51 (Figure 5A) were identified as titratable. CpHMD simulations were then performed to obtain the titration curves of H12, E45, and D51 in the presence or absence of IP6 for each of the six CA subunits that form the hexamer. The titration curves for the pentapeptides AAXAA (where X = H, E, and D) were calculated and used as references (Figure S6). The CpHMD simulations of the CA hexamer covered a pH range between 3.2 and 8.4, and the total simulation time for each setup was 5.6 μs.

**Figure 5:**
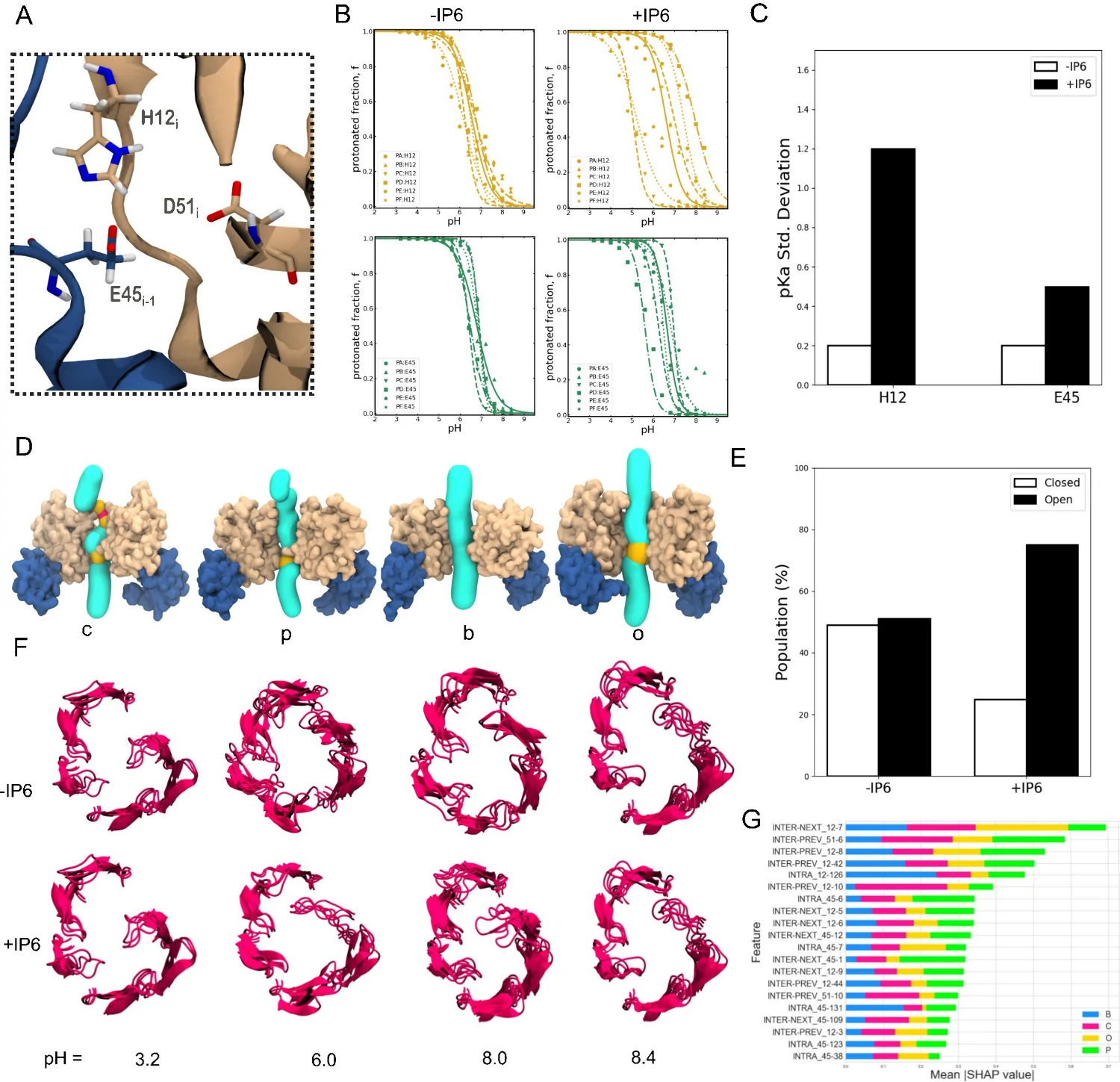
**CpHMD simulations of CA hexamers in the absence and presence of IP6.** (A) Structure of assembled CA highlighting the interface between subunits, which are differential colored to clearly identify different subunits. Residues H12_*i*_ and D51_*i*_ from CA subunit *i* (tan) are shown in licorice. Residue E45_*i*+1_ from CA subunit *i* + 1 (navy) is shown in licorice. (B) Titration curves from H12 (gold) and E45 (green) in the absence (left) and presence (right) of IP6. (C) Standard deviation of calculated p*K*_a_ for the six CA subunits within the CA hexamer. White bars correspond to the absence of IP6 and black bars to the presence of IP6. (D) HOLE analysis of the pore pathway. From left to right closed (c), protruding (p), broken (b), and open (o) pores are shown. Pore class is indicated at the bottom of the hexamer image. The pathways for each class are colored by the calculated radii: 1.5 Å (size of a water molecule) is magenta, 3.5 Å (length of dNTP) is yellow, and *r* > 3.5 Å is cyan. The first constriction point at the top of the channel is where the β-hairpins are found. The second noted constriction point is at R18, which is known to coordinate IP6 but is not related to the characterization of the classes. (E) Percentage of closed (white bars) and open (black bars) pores in the absence (left) or presence (right) of IP6. (F) Structures of PAM cluster-representative β-hairpin hexamers. Absence (upper row) and presence (bottom row) of IP6 are indicated. The pH (3.2, 6.0, 8.0, and 8.4 from left to right) is indicated at the bottom-left side of each structure. (G) Mean SHAP values indicating the importance of specific contacts for the c, p, b, and o pore states. On the left side, the contacting residues and whether the interaction is mediated in a single CA or corresponds to inter-CA–CA contacts is specified. The contacts within protomer *i* are represented as INTRA; those between protomer *i* and protomer *i* – 1 are represented as INTER_PREV; those between protomer *i* and protomer *i* + 1 are represented as INTER_NEXT.

In the absence of IP6, the p*K*_a_ values of the 18 residues H12, E45, and D51 corresponding to the six chains were determined by fitting the titration curves to the Henderson–Hasselbalch equation and compared with those of the reference model pentapeptides (Table 2 and Figure 5B). For H12, the mean p*K*_a_ was found to be similar to that obtained for the pentapeptide (6.5 ± 0.2 and 6.4, respectively). While the p*K*_a_ for E45 significantly upshifted from the pentapeptide (6.7 ± 0.2 vs 4.5), the p*K*_a_ of D51 is lower than the minimum pH value explored in our simulations, and the p*K*_a_ calculated is downshifted from the value observed for the pentapeptide (<3.2 vs 4.1). In the presence of IP6, while the average p*K*_a_ was maintained, interchain variability increased, especially for H12 (Table 2 and Figure 5B,C). This suggests that IP6 enhances protonation-state heterogeneity of the local network. For D51, no changes to the protonation state are observed either at pH close to physiological or within the experimental range used here. The critical role of the P1–D51 salt bridge is underscored by experimental evidence showing that mutation to alanine results in a mixture of cylindrical and amorphous assemblies observed by TEM, reduced RT activity, and complete loss of infectivity.^38,41^ In addition, IP6 binding introduces a significant increase in the p*K*_a_ standard deviation for H12 and, to a lesser extend in E45, which suggest protonation heterogeneity across CA subunits within the hexamer.

**Table 2:** Calculated p*K*_a_ Values of Reference Pentapeptides and of 18 Titratable Residues in the Hexamer in the Absence and Presence of IP6

	H12		E45		D51	
	–IP6	+IP6	–IP6	+IP6	–IP6	+IP6
reference	6.4		4.5		4.1	
chain A	6.2	7.4	6.9	6.5	<3.2	<3.2
chain B	6.6	6.6	6.8	6.6	<3.2	<3.2
chain C	6.7	5.0	6.4	7.0	<3.2	<3.2
chain D	6.8	8.0	6.5	5.6	<3.2	<3.2
chain E	6.5	5.2	6.8	6.8	<3.2	<3.2
chain F	6.3	7.0	6.9	6.3	<3.2	<3.2
average	6.5 ± 0.2	6.5 ± 1.2	6.7 ± 0.2	6.5 ± 0.5	<3.2	<3.2

To determine whether the observed p*K*_a_ variability reflects genuine heterogeneity or insufficient sampling, we analyzed cumulative and block-averaged p*K*_a_ estimates for each chain. In the absence of IP6, all six H12 residues converge to similar p*K*_a_ values (6.5 ± 0.2), with minimal variability between chains (Figure S7). In contrast, under IP6-bound conditions, the six chains converge to distinct and persistent p*K*_a_ values, while within-chain variation remains much smaller than the observed inter-chain spread (Figure S8). Together, these results demonstrate that the variability arises from IP6-induced structural heterogeneity rather than from inadequate sampling or limitations of the CpHMD protocol.

In the IP6-bound simulations, IP6 was assigned a formal charge of −12 because direct IP6 titration is not currently supported in CpHMD. Comparison with a variable-charge model (−6/–8/–10; see Methods) yielded similar H12 and E45 p*K*_a_ values and preserved their placement within the physiologically relevant pH 6–7 range (Table S2). Stable R18–IP6 contacts across pH 3.2–8.4 further support the validity of the fixed-charge approximation. Thus, the qualitative conclusions are unaffected by the IP6 charge model, and all subsequent analyses were carried out using the −12 charge state.

### Binding of IP6 to CA Hexamer Pores Rewires a pH-Dependent Network to Alter Pore State Distributions

We sought next to identify potential links between conformation and function under different conditions, including different pH and the absence or presence of IP6. To this end, PAM clustering was applied using an in-house algorithm^42^ to identify representative conformational subsets of β-hairpins in the assembled hexamers (Figures 5F, S9, and S10). The analysis revealed that hexameric pores adopt a range of conformations during simulation, generally favoring elliptical rather than perfectly circular shapes. Structural inspection was combined with channel radii measurements using HOLE2 ^43^ (Figures S11 and S12) to characterize the cross-sectional geometry of the pore channel under different conditions. Based on the full HOLE2 pathway profile and PAM clustering of β-hairpin conformations, four different pore classes were found, both in the absence and in the presence of IP6: class 1 (closed pore), geometrically incompatible with the dNTPs passage; classes 2 and 3 (protruding and broken), whose geometric compatibility with dNTPs passage remains uncertain; and class 4 (open pore), which has a rather retracted pore, that is geometrically compatible with the nucleotide entry (Figures 5D and S13). The dNTP molecule (∼6 Å in length) was used as a qualitative reference to assess structural compatibility of open conformations with nucleotide entry based on the cross-sectional radii. We note that actual dNTP passage depends on multiple factors including cross-sectional radius, solvation, electrostatics, and conformational dynamics not captured by the geometric measurements alone. The overall populations for each category in the presence and absence of IP6 indicate that there are comparable amounts of each class (Figure S13E). Assuming with caution that the closed and open states identified by CpHMD simulations may not correspond exactly to those identified by HS-AFM, we have made a comparison between both results. If both results are consistent, we might think that the simulations are faithfully reflecting the results determined experimentally, which strengthens the conclusions we can find by using both approaches in combination. In the absence of IP6, the results of the CpHMD simulations yielded a close:open ratio (49:51 %) consistent to that determined in HS-AFM experiments (64:36 %). Likewise, in the presence of IP6, the simulations yielded a closed:open ratio (20:80) that matched the ratio determined in HS-AFM experiments (20:80) (Figure 5E and Table 1). This agreement suggests that the MD simulations adequately reproduced the experiment, encouraging the use of such simulations for detailed structural analysis of the conformational changes involved in the opening of the pores promoted by IP6 binding or acidification.

Having established the pore classes, classification machine learning algorithms, and extreme gradient boosting (XGB),^44^ were employed to predict key residue pairs that distinguish each pore class. In the absence and presence of IP6, 143 and 126 center of mass (COM) distance features were extracted with VMD. The feature space was defined using pairwise distances between side-chain centers of mass contacting one of the titratable groups, thereby constructing a contact-based network that captures conformational changes across the pH range. Feature importance was assessed with the implementation of Shapley additive explanations (SHAP)^45^ (Figure 5G). SHAP values quantify the contribution of each feature toward the classification of each pore class. The model was trained and evaluated using train/test divisions 90/10, 80/20, and 70/30 of the full data set to assess its generalizability and check for potential overfit. From this evaluation, only 70 features were required to maintain the accuracy of the full data set. For the selected subset of features, the confusion matrices represent the performance of the model as a function of the pore state both in the absence and in the presence of IP6 (Figures S14 and S15, respectively). The distribution of subset COM features in the order of importance was then plotted across the full pH range to assess pH-dependent shifts in the corresponding states. The SHAP *beeswarm* plots were then constructed for each feature representing their contribution, where the color of the distribution corresponding to positive values indicates the magnitude of the feature contributing to the classification. To reach 80% accuracy, it was necessary to consider 20 and 70 features for the absence or presence of IP6, respectively. Residue pair COM distance distributions of the top-ranked features were then plotted to assess whether pore dynamics exhibit pH dependence in the absence (Figure S16) or presence (Figure S17) of IP6. Residue pairs relevant for characterizing the pore state in the absence of IP6 include H12_*i*_–M118_*i*_, H12–T119_*i*_, and D51_*i*_–P1_*i*_ in the same chain and E45_*i*_–D51_*i*+1_, E45_*i*_–P17_*i*+1_, and E45_*i*_–L20_*i*+1_ between CA subunits. It is interesting to note that E45 establishes contacts with P17 and L20, which are linked to R18, a critical residue in the IP6 binding site, suggesting a link between E45 and R18 that is more prominent under acidic conditions and is turned off upon binding IP6. Interestingly, among the distance pairs established by the E45–H12–E45 interaction, it emerged as the most significant feature for pore classification in the absence of IP6. As many as ten residue pairs exclusive to the IP6-bound state were identified as relevant to determining the pore state. These include E45_*i*_–A47_*i*_, H12_*i*_–Q50_*i*_, and H12_*i*_–L111_*i*_ in the same CA subunit and H12_*i*_–V3_*i*–1_, H12_*i*_–N5_*i*–1_, H12_*i*_–G8_*i*–1_, H12_*i*_–M10_*i*–1_, E45_*i*_–P1_*i*+1_, E45_*i*_–Q50_*i*+1_, and E45_*i*_–T58_*i*+1_ between CA subunits.

IP6 binding promotes H12 interactions with residues from neighboring β-hairpins (i.e., V3, N5, G8, and M10), causes E45 to lose its connection to the IP6 binding site via P17 and L20, introduces a new inter subunit E45 contact with P1 and cements the D51-P1 salt bridge that is allowed to locally release without IP6 present. D51 does not exhibit pH-dependent behavior in the presence of IP6; however, in the absence of IP6, both D51_*i*_–P1_*i*_ and E45_*i*_–D51_*i*+1_ demonstrate a pH sensitivity. Overall, the results highlight how IP6 stabilizes the network around D51, whereas its absence allows D51 to engage in pH-dependent contacts.

### Binding of IP6 to the CA Protein Lattice Increases Both Lattice Stiffness and Resistance to Material Fatigue

We have previously shown that the physical properties (flexibility and strength) of the HIV-1 CA protein lattice can be modified by binding small molecules, including some with antiviral activity.^46^ In the present study, we investigated whether the proviral factor IP6 may also modulate the mechanical properties of the CA protein lattice. To this end, the stiffness, strength against disruption by point forces, and resistance to material fatigue of the CA lattice in the absence or presence of IP6 were compared.

To determine the effect of IP6 on the stiffness of the CA protein lattice, its spring constant *k*_s_ was determined by shallow indentations using an AFM instrument (Figure S18A). The results (Table 3) showed that the *k*_s_ value in the presence of IP6 was 18 % higher than in its absence (Figure 6A). The effect of IP6 on the lateral mobility of the CA lattice, taken as an indicator of lattice stiffness in the plane, was determined by measuring the breathing amplitude (*A*_b_) (Figure S18B), which is the amplitude of the relative movements of individual hexamers within the CA lattice.^31^ The results (Table 3) showed that in the presence of IP6 the *A*_b_ value was 31 % lower than in its absence (Figure 6B). These results indicate that binding of IP6 to the hexamer pore rigidifies the CA lattice in three dimensions.

**Table 3:** Mechanical Parameters of the CA Lattice in the Absence or Presence of Bound IP6

	*k*_s_			*A*_b_		*F*_r_			*t*_50_		
	av. ± s.d. (N/m)^a^	*N* ^b^	*p* ^c^	*A*_b_ ± err. (nm)^d^	*N* ^b^	av. ± s.d. (nN)^a^	*N* ^b^	*p* ^c^	av. ± s.d. (min)^a^	*N* ^b^	*p* ^c^
–IP6	0.43 ± 0.10	39	–	1.15 ± 0.07	231	1.26 ± 0.21	52	–	24 ± 10	8	–
+IP6	0.51 ± 0.10	85	1.4 × 10^–7^	0.79 ± 0.01	180	1.41 ± 0.31	37	3.1 × 10^–2^	49 ± 24	7	3.5 × 10^–2^

a Average value ± standard deviation.

b Number of measurements.

c *p* value of one-tailed Student’s *t* test using α = 0.05.

d Value and fitting error obtained by adjusting the distribution of interhexamer distances to a Gauss function (2).

**Figure 6:**
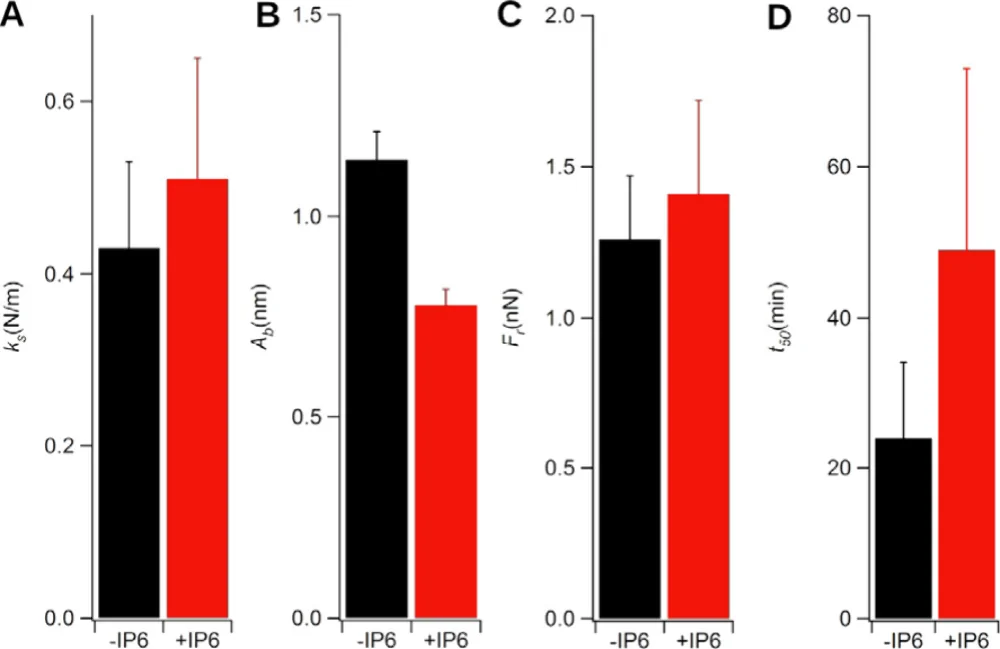
**Mechanical parameters of the CA protein lattice in the absence or presence of bound IP6.** Bar plots show (A) the spring constant *k*_s_ (average value ± s.d.), (B) the breathing amplitude *A*_b_ (fitting value ± fitting error), (C) the rupture force *F*_r_ (average value ± s.d.), and (D) the half-life for disassembly *t*_50_ subjected to material fatigue (average ± s.d.). The black bars correspond to values obtained in the absence of IP6, and the red bars correspond to values obtained in the presence of IP6.

To determine the effect of IP6 on the resistance of the CA protein lattice against disruption by a point load, deeper indentations were performed (Figure S18C). Previous results had shown that most indentations of the CA lattice led to two disruption events. The second event led to the complete disruption of the lattice at the point indented.^31^ In the present study, the values of the force *F*_r_ required for the second disruption event in the presence or absence of IP6 were compared (Table 3 and Figure 6C). IP6 binding to the CA lattice led to a moderate but statistically significant increase in mechanical strength (average Δ*F*_r_ = 11%).

Finally, to determine the effect of IP6 on the resistance of the CA protein lattice to disruption by material fatigue, experiments were carried out as described in Methods. The time required for disruption of half the indented area under the conditions tested (*t*_50_) was taken as a measurement of the relative resistance to fatigue. The results (Table 3 and Figure 6D) showed that binding of IP6 drastically increased the resistance of the CA lattice to fatigue (average Δ*t*_50_ = 104%).

### Integrated Model of the Role of IP6 during HIV-1 Transcription

The combined use of HS-AFM and SPA has great potential for studying the conformational dynamics of individual molecules near equilibrium, as demonstrated in the present study, which has been focused on the dynamics of individual hexamers in the HIV-1 capsid protein lattice. Despite previous work on the role of IP6 during HIV-1 transcription, a detailed dynamic view from the single-molecule perspective is missing. In this work, we also combined HS-AFM measurements with all-atom MD simulations and mechanical measurements to investigate, at the single-residue level, the structural changes elicited by IP6 that could promote the opening of the capsid pores. Overall, the results presented in this work support a mechanism by which binding of IP6 to the HIV-1 core facilitates reverse transcription of the viral genome during its transport to the cell nucleus.

The high temporal resolution of the HS-AFM data revealed a highly dynamic behavior of the hexamers in the CA protein lattice. The CA hexamers transiently showed a conspicuous protrusion (bulge) on the 6-fold symmetry axis where the pore is located. A transition occurs at equilibrium between two conformational states of the CA hexamer: state 1, in which the bulge is present, and state 2, in which the bulge is absent. These two states were populated in different proportions, depending on the conditions: absence or presence of IP6, acidic or basic pH. Since the bulge was also observed on many hexamers in the absence of IP6, it could not correspond to the IP6 molecule itself. Given the absence of a clear correlation between the single-molecule data obtained in this study and the average structural data previously available, we hypothesize that the bulge may arise from the unfolding of one or more of the six β-hairpins that surround the pore.

SPA results described above indicated that state 2 corresponds to hexamers in which the pore is presumably open (O state), and state 1 corresponds to hexamers in which the pore is presumably closed (C state). Previous work based on averaged data had suggested that at pH < 7 the pore would be open, while at pH > 7 it would be closed.^15^ However, the single-molecule data presented here revealed that the transition caused by a pH change is not that sharp. About half of the individual pores were found to be in one of the two states at pH 8, while virtually all of the individual pores were found in the open state at pH 6. These data show that the pH sensor, located at the pore entrance, is not as sensitive under slightly basic pH compared to slightly acidic pH. This effect could be due to the fact that, under basic conditions, a water molecule is needed to mediate the interaction between the β-hairpin and helix α1, whereas under acidic conditions this is not the case. Since an indirect interaction may be less stable, the β-hairpin could transition more easily between the two conformations at pH 8. The results obtained here suggest that both acidification and IP6 binding could promote the opening of the pore through a change in the p*K*_a_ of the H12 residue.

By determining the relative number of individual pores in either state, the free energy difference between the two states was calculated (see the Supporting Information). The small stabilization by IP6 of the open state relative to the closed state at pH 8 suggests that only subtle conformational changes, such as β-hairpin annealing and pore deconstriction are involved in the pore opening transition. Equally small free energy differences have been found associated to the opening of ion channels.^47^ Although the free energy difference between the closed and open states might be small, the pore opening kinetics, namely the activation free energy barrier, may be most important for controlling pore opening, dNTP translocation and RNA retrotranscription during HIV-1 infection. The SPA approach using HS-AFM images could be used to determine the dwell times of the closed and open states, and from this data, the activation free energy for pore opening transition. However, to perform the proposed kinetic study it will be necessary to first develop a dedicated Scipion plug-in (work in progress).

The different p*K*_a_ shifts of residues H12 and E45 relative to those in a free, unfolded peptide, when different subunits in the same hexamer were compared, reflect a different local structural context in each CA subunit. Interestingly, a different local structural context was previously revealed by different R18 orientations of subunits in assembled CA tubes.^40^ Similarly, the cluster analysis performed here indicated that the CA β-hairpins are asymmetrically arranged in the presence of IP6 or at low pH. We hypothesize that both IP6 and a low pH promote the opening of the pores through a similar mechanism. When IP6 binds the hexamer, H12 and E45 would rewire their contact network in a single subunit at first. Then, the conformational change would propagate to neighbor subunits through β-hairpin interactions, and the end result would be a fully open pore in which the six β-hairpins are in the same conformation. Propagation of conformational changes upon molecule binding to symmetric protein complexes has been identified as a reliable mechanism for allostery,^48,49^ also in other viral systems.^72^ It is worth noting that residue D51 does not exhibit pH-dependent behavior in the presence of IP6; however, in the absence of IP6, both the D51_*i*_–P1_*i*_ and E45_*i*_–D51_*i*+1_ interactions display clear pH sensitivity. The prevalence of the D51–P1 contact behavior is attributed to the observation that the β-hairpin unfolded in a single protomer at pH 4.0 without IP6 in this study, which can be considered a low probability event. The unfolded β-hairpin behaves as an intrinsically disordered region,^50^ interacting transiently with the CypA binding loop or CA interfaces rather than adopting a rigid upward configuration.

The ongoing research on the mechanical properties of viruses has already highlighted the importance of viral particle stiffness and strength to optimize infection.^51^ The elasticity and resistance to disruption by point forces of the mature HIV-1 core have been determined by AFM.^8,52,53^ However, those measurements were obtained using a complex, composite nucleoprotein particle engaged in reverse transcription of the internal RNA. In the present study we used instead just the hexagonal CA protein lattice as a model to specifically determine the effect of IP6 on the fundamental protein “fabric” that makes the mature HIV-1 capsid. The results obtained showed that IP6, in addition to promoting pore opening, stiffens the CA lattice, in agreement with previous all-atom molecular dynamics studies showing that IP6 enhances capsid rigidity.^54^ The two effects may be related, as stiffening the lattice may help retain the CA β-hairpins in the conformation required for keeping the pores open. The IP6-mediated stiffening of the CA lattice may contribute to impair biologically nonproductive conformational changes. Previous studies had shown that IP6 biochemically stabilizes the HIV-1 capsid.^9,11,12,26,55^ Previous work also indicated that the mechanical stability of the HIV-1 core is linked to the infectivity of the virion.^56–58^ The present study has revealed, in addition, that IP6 also increases the resistance of the CA lattice to material fatigue. This stabilization may also be biologically relevant by helping the HIV-1 core to withstand low-force but frequent collisions with other particles and macromolecules in the crowded environment of the infected cell.^52,59,60^ These results suggest that, in contrast to capsid-stabilizing compounds such as lenacapavir that bind inter-subunit interfaces,^61^ targeting the hexameric pore, also modulated by molecules like hexacarboxybenzene^15^ offers a complementary strategy to inhibit reverse transcription by restricting nucleotide import, with potential for synergistic antiviral effects.

The ensemble of results indicates that IP6 exerts its proviral effect at two complementary levels: (i) promoting pore opening, and (ii) chemically and mechanically stabilizing the capsid protein lattice. The results allow us to propose the following model to explain the proviral activity of IP6 (Figure 7):

(i). IP6 binding to the mature HIV-1 capsid increases the contacts between CA β-hairpins in an allosteric manner, placing the six β-hairpins in a hexamer in a conformation compatible with the open pore state.
(ii). At the concentration of IP6 used here, most (80%) of the pores were found in the open state. Thus, it could be expected that in the cytosol, where the concentration of IP6 is much higher,^12,62^ nearly all of the pores in the mature core would be in the open state, maximizing dNTP translocation probability into the capsid and promoting a fast reverse transcription of the viral genome.
(iii). Intracellular mechanics^63^ might be paramount when the intracellular transport of viral capsids is considered. An IP6-induced increase in the resistance to material fatigue of the HIV-1 core being transported on microtubules^64^ would be biologically advantageous under the low-force but frequent collisions it may experience in the macromolecularly crowded intracellular environment.

**Figure 7:**
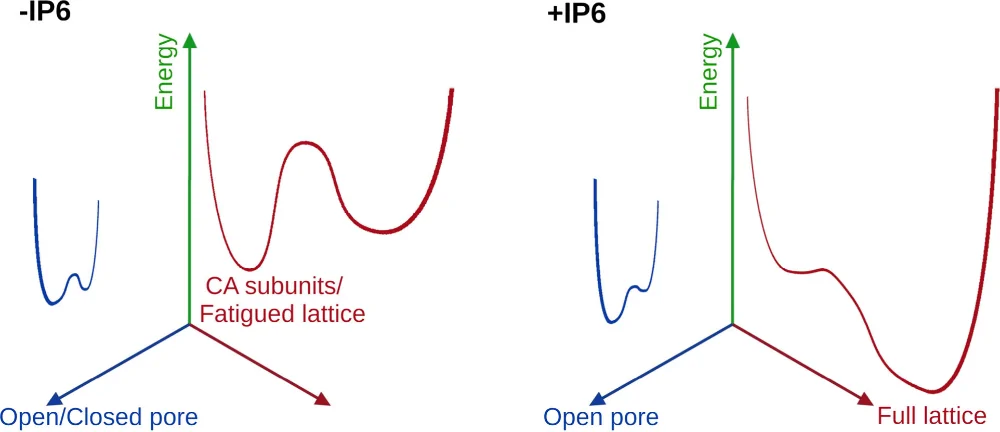
**Proposed model integrating IP6 activity at two levels: pore opening and lattice stabilization.** An energy scheme (green axis) is considered that takes into account the assembly/disassembly of the hexagonal lattice under material fatigue (red axis) and the opening of pores (blue axis) as orthogonal reaction coordinates. In the absence of IP6 (left) and under mechanical fatigue, the CA lattice becomes energetically destabilized, leading to breaking or disassembly of the lattice into CA subunits. In this case, for the pores present in the lattice, the open and closed states are similarly populated (indicated as two wells in blue). In the presence of IP6 (right), both the complete integrity of the lattice and the open pores are energetically favorable (left blue and right red wells).

## Conclusions

This study explores the effects of IP6 on conformational states of the pore and on the mechanical properties of the mature HIV-1 capsid protein lattice. Using a novel approach combining high-resolution HS-AFM movies and SPA, we visualized in real time individual capsid pores in the closed or the open state and determined the pore state population. By performing CpHMD simulations, we found that the binding of IP6 to the CA lattice reconfigures the CA–CA contacts, propagating a conformational change between the subunits that leads to a higher probability of finding open pores, which may facilitate the translocation of dNTPs for the reverse transcription of the viral genome. In addition, AFM-based mechanical analysis revealed that IP6 increases the stiffness and resistance to material fatigue of the CA lattice. An increased stiffness would limit the biologically unproductive closure of the pores, and an increased fatigue resistance would help the HIV-1 core withstand frequent collisions in the crowded intracellular environment during transport to the cell nucleus.

## Methods

### Protein Expression and Purification

A plasmid (pET21b­(+)) containing the coding sequence for CA from HIV-1 (strain BH10) was used to express the HIV-1 capsid protein CA in *Escherichia coli* BL21­(DE3).^65^ Cell cultures were grown at 37 °C and protein expression was induced by adding 1 mM IPTG and incubating for 3 h. Cells were collected and frozen at −20 °C for storage. The cells were thawed and resuspended in 50 mM Tris-HCl (pH 8) with 5 mM β-mercaptoethanol and lysed by sonication. After centrifugation, the CA protein from the soluble fraction was purified by ammonium sulfate fractionation, ion exchange chromatography on SP-Sepharose, and size exclusion chromatography on Superdex 75 (Amersham Biotech). The purified CA solution thus obtained was completely dialyzed against phosphate-buffered saline (PBS) and stored at −80 °C until use. Protein purity was assessed by polyacrylamide gel electrophoresis. The concentration of CA protein stocks was determined by UV spectrophotometry.

### CA Protein Lattice Self-Assembly for HS-AFM Imaging

The purified CA protein stored in PBS (pH 7.4) was diluted with 50 mM phosphate buffer, 137 mM NaCl at pH 7.4 to a final CA concentration of 1 μM, and incubated on a freshly cleaved mica substrate for 1 h at room temperature (r.t.) (25°C). Next, 50 mM phosphate buffer, 137 mM NaCl at pH 8 or 6 was added to replace the previous buffer. For experiments with IP6, a buffer containing 1 mM IP6, 50 mM phosphate buffer, and 137 mM NaCl at pH 8 was added to the liquid cell and incubated for 30 min at r.t.

### HS-AFM Imaging

Ultrashort cantilevers (USC-F1.2-k0.15-10, NanoWorld, with the tip grown on top of the cantilever by electron beam deposition and sharpened by plasma etching)^66^ with a nominal spring constant of 0.15 N/m were automatically calibrated with the JPK NanoWizard SPM software (Bruker) after mounting into the high-speed atomic force microscope (NanoRacer, Bruker). Imaging with the HS-AFM was performed in amplitude modulation mode with the minimal necessary reduction in the oscillation amplitude (10-20 %) to engage the sample. The size of the images was fixed (50 nm × 50 nm) in order to properly visualize about 25 hexamers in a single image by using 128 × 128 or 256 × 256 points per image. All reported AFM images were acquired with an imaging time ranging from 0.9–1.3 s/frame. The JPK SPM data processing software (Bruker) was used minimally to flatten and export videos for later calibration with ImageJ.^67^

### Simulation of AFM Images and Topographic Profiles

AFM images and corresponding topographic profiles were simulated using BioAFMviewer^68^ and PDB entries 6b2j and 6b2i for the closed and open pores of the E45A CA mutant, respectively. These pdb files were chosen because of the large differences in the angle of the β-hairpin and the diameter of the pores between open and closed pores (Figure 1). The used simulation parameters were: scan step of 0.47 nm (experimental scan size divided by the number of scan points), cone angle of 5°, and tip radius of 2 nm. These simulated AFM images are based on static structural models and simplified tip–sample interaction assumptions, and therefore provide for a qualitative rather than quantitative comparison with experimental AFM data.

### Single Particle Analysis of CA Hexamers

For structural classification, movies processed with ImageJ^67^ were divided into individual frames and imported using the Scipion cryo-EM processing framework.^34^ First, approximately 75 hexamers were manually selected using a frame size of 12 x 12 nm. If this size was reduced or increased, the software could not automatically detect the hexamers afterward. Next, automatic particle selection was used to extract hexamers as individual particles from the movies. Manual and automatic particle selection and the extraction of individual particles (hexamers) were performed using *xmipp3*.^69^ Once all hexamers had been extracted, several rounds of classification (Figure S1 and Table S1) were performed using *cl2d*.^35^ The average image of all hexamers belonging to the same class was obtained and used to eliminate classes that showed incomplete or low-resolution hexamers. Given the nature of the classification process, discarding or accepting classes is a user-dependent process. To minimize any user-dependent bias, refinement cycles were carried out in parallel by two users, ultimately obtaining a similar number of hexamers showing the pore in one of the two main states identified, which changes its population under each condition tested. Between 8 and 24 classes were used during classification refinement, as described above. Finally, 6 classes were used in the final step, as using fewer classes caused the algorithm to begin mixing hexamers that clearly belonged to different classes. Once the classes corresponding to open or closed pores were identified, they were grouped together and the averages for open and closed pores were obtained for every condition tested.

### Self-Assembly of CA Protein for Mechanical Analysis

Self-assembly of the purified CA protein lattice on a freshly cleaved mica surface was carried out at r.t. in PBS under physiological conditions of ionic strength and pH.^46^ The protein concentration for assembly was 0.7 μM or 1 μM for fatigue measurements or other mechanical measurements, respectively. The coverage of the assembled lattice was close to 100 % after approximately 1–1.5 h of incubation. Once the assembly reaction was complete, the protein monolayer was subjected to several washes with PBS to remove any remaining protein in solution. AFM imaging was used to check both the architecture and dimensional parameters of the assembled lattice and to quantify the substrate coverage.

### Mechanical Analysis of the CA Protein Lattice by AFM

For measurements of lattice flexibility and strength, an atomic force microscope (Nanotec Electrónica, S.L.) controlled by WSxM software was used in Jumping Mode.^70^ For each experiment, the actual spring constant of the qp-BioAC (0.1 N/m nominal spring constant, Nanosensors) cantilevers was determined by using Sader’s method.^71^

#### Determination of Stiffness and Breakage Force

The spring constant of the CA protein lattice, *k*_s_, in the absence or presence of IP6 were determined by performing shallow indentations (≤2 nm) to remain within the elastic regime and considering two springs, sample and cantilever, in a series.^46^ To induce lattice breakage and determine the breakage force, larger displacements (>20 nm) were performed until the cantilever tip came into contact with the rigid mica substrate. Mechanical rupture of the HIV-1 lattice using AFM typically reveals two rupture events before the lattice breaks;^31^ the final event—the one requiring the greatest force—indicates mechanical strength under point loads and is therefore the one we have considered in this study, *F*_r_.

#### Determination of the Breathing Amplitude

Large CA protein lattice patches were imaged at high spatial resolution by AFM, either in the absence or presence of IP6, to determine the relative positions of hexameric subunits in the lattice and the breathing amplitude.^31^ A single hexamer frame was used as template to recover the positions of each hexamer within the full image by using the Template Matching plugin^72^ in the Fiji (ImageJ) software.^67^ Then, first neighbor hexamer–hexamer distances were calculated, and the probability density function was plotted. To determine the breathing amplitude, *A*_b_, the distance distribution was fitted to the following Gaussian function:

1 $${y=y}_{0}+D\cdot \exp\left(-\frac{2\cdot \left(b-b_{0}\right)}{A_{b}}\right)$$

where *y* corresponds to the probability density, *y*_0_ is the baseline, *D* is the height of the curve’s peak, *b*_0_ is the lattice parameter in equilibrium, and *b* is the actual hexamer to hexamer distance.

#### Determination of the Resistance to Material Fatigue

The resistance of the CA protein lattice to disintegration by material fatigue in the absence or presence of IP6 was determined as described next.^46^ Full coverage CA lattice was continuously imaged (200 nm × 200 nm and 128 × 128 pixels) in Jumping Mode at a maximum force of 60 pN at a frequency of 0.7 min^–1^ until the CA lattice had been completely disassembled. To calculate the percentage surface area covered by the remaining CA lattice, SA, as a function of time, *t*, the Flooding Tool included in WSxM^70^ was used, and the data were fitted using the Hill function:

2 $$\mathrm{SA}={\mathrm{SA}}_{0}+\frac{{\mathrm{SA}}_{F}-{\mathrm{SA}}_{0}}{1+{\left(\frac{t_{50}}{t}\right)}^{n}}$$

where SA_0_ and SA_F_ are the initial and final surface areas covered by the lattice, respectively; *t*_50_ is the parameter taken to determine the resistance to material fatigue and corresponds to the half-life of the capsid lattice in which the surface area covered was reduced by 50%; and *n* is a cooperative index.

### Constant-pH Molecular Dynamics Simulations

#### System Setup

The initial structure for constant-pH molecular dynamics (CpHMD) simulations was obtained from previous equilibrated (2 μs) CA hexamers in the presence and absence of IP6 and presenting closed or open pores.^9^ Parameters for IP6 were derived from the CHARMM General Force Field (CGenFF).^73^ Hexameric complexes were solvated with TIP3P^74^ water model and ionized to a final NaCl concentration of 150 mM by using VMD.^75^ For constant-pH simulations of the IP6 bound structure, the model after the full 2 μs was chosen, however, with no IP6, we observe a conformational change of the pore corresponding to the change in the RMSD over time at 250 ns, where a β-hairpin is observed to shift to a closed state. To minimize model bias, the constant-pH assay in the absence of IP6 was initiated from the structure prior to 250 ns.

#### Constant-pH MD Simulations

CpHMD simulations were carried out using NAMD 3.0 ^76^ and the CHARMM36m force field,^77^ using a hybrid nonequilibrium MD/Monte Carlo (neMD/MC) framework.^36,78^ Using the CpHMD method, at regular intervals during simulation, depending on the chemical environment and the chosen pH, Monte Carlo calculations are used to evaluate the protonation states of the titratable sites. This method uses neMD/MC cycles using a λ-dynamics formalism, where the protonation coordinate λ varies continuously between 0 ≤ λ ≤ 1 (0 = deprotonated, 1 = protonated). The λ coordinate is integrated alongside the system’s physical degrees of freedom, enabling smooth protonation-state transitions.

The residues H12, E45, and D51 in each CA subunit of the hexamer were selected for titration, resulting in 18 titratable groups. Each CpHMD assay was carried between pH values from 3.2 to 8.4 with a step size of 0.4 to sweep through range of pH values encompassing acidic, basic and physiological conditions.^79^ Each cycle in the CpHMD consisted of 5000 steps (10 ps) of equilibrium MD, followed by a protonation switching attempt. Nonequilibrium simulations were then propagated for 7500 steps (15 ps) in a dual-topology representation, smoothly coupling protonated and deprotonated states. Acceptance or rejection of the switch was determined using the nonequilibrium work criterion in the Metropolis criterion followed by a 5000-step minimization before returning to equilibrium MD. A total of 40,000 neMD/MC–MD cycles were carried out at each pH, yielding ∼5.6 μs of MD sampling. The simulation temperature was set to 310 K controlled via Langevin dynamics with a damping coefficient of 1 ps^–1^.^80,81^ The pressure was maintained at 1 atm. Long-range electrostatics were treated with the particle mesh Ewald (PME)^82^ method at a grid spacing of 1.0 Å. The van der Waals interactions were smoothly switched between 10 and 12 Å, with a 14 Å pairlist cutoff. Rigid bond constraints were applied to all bonds involving hydrogen atoms.^83,84^ Titration curves were computed using the *cphanalyze* utility in the PyNAMD package.^85^ Protonation-state populations were analyzed with the unbinned weighted histogram analysis method (WHAM),^86–88^ which reweights data collected across the evaluated pH range. Because the Hamiltonian is pH-independent, the WHAM equations reduce to functions of the λ coordinates, yielding analytic titration curves. The titration curves were fit to the Henderson–Hasselbalch equation to determine apparent p*K*_a_ values and Hill coefficients. For analysis, the first 2000 CpHMD cycles were discarded. IP6 is considered with a formal charge state of −12 across all the pH. To assess the convergence of the H12 and E45 p*K*_a_ estimates, each trajectory was divided into four independent blocks (iterations 0 to 4, 5 to 9, 10 to 14, and 15 to 19, each comprising 2000 neMD/MC cycles) (Figures S7 and S8). The p*K*_a_ values were computed independently per block per chain without any overlap. p*K*_a_ convergence was also assessed using cumulative pooled sampling windows (iterations 0 through *i*, corresponding to 2000–40,000 neMD/MC cycles per pH condition) (Figures S7 and S8). Reference pentapeptide systems AAXAA (X = ASP, GLU, or HIS) were equilibrated for 30 ns. The following pH ranges were used: 1.5–5.0 (ASP), 2.5–6.5 (GLU), and 4.5–8.0 (HIS) with a step size of 0.5. Each cycle consisted of 10 ps MD and 15 ps neMD/MC over 2000 cycles, for an aggregated sampling of 160 ns for each of the three pentapeptide systems. The other parameters for the CpHMD assays were retained as in the hexamer systems. To account for the possibility of different charge state of IP6 across the pH range used in the study, CpHMD simulations were also carried out for 10,000 cycles with varying IP6 protonation states. At pH 4.4–6.0, a charge state of −6, at pH 6.4 to 8.0, a charge state of −8, and at pH 8.4, a charged state of −10 were used. p*K*_a_ values were estimated similar to the prior simulations by discarding the first 2000 cycles. The formal charges were assigned to each pH range based on the reported studies on phytic acids at varying pH conditions.^89,90^

#### Pore-State Characterization

An in-house PAM clustering algorithm^42^ was employed to define the conformational subspaces in each pH simulation. Silhouette scores were used to determine an appropriate number of clusters via local maximums.^91^ Four categories of pores were identified by visual inspection and analysis with HOLE2.^43^ Classification machine learning was applied to extract the structural features that distinguish each pore class by implementing Shapely additive explanations (SHAP).^92^ To this end, pairwise side-chain center of mass (COM) distances found closer than 7 Å with titratable residues (H12, E45, and D51) were extracted from the CpHMD simulations using VMD. Defaults within PyCaret classification pipeline with extreme gradient boosting (XGBoost)^93^ were selected. SHAP values were used to identify the top features and evaluate a minimal subset of features required for the model. While most features did not exhibit a particular pH-dependent contact (within 7 Å) with one of the targeted titrable groups, 18 were identified in the presence of IP6, while 13 were identified in the absence of IP6. The identified residues were then represented at the CA–hexameric interface to demonstrate the influence of IP6.

## Supplementary Material







## Data Availability

The data that support the findings of this study are available from the corresponding authors upon reasonable request.

## Notes

Table including the number of HS-AFM movies analyzed, particles extracted, and classes used in different steps during classification (Table S1); table comparing p*K*_a_ values of H12 and E45 residues within different IP6 charge assignments (Table S2); scheme showing the workflow followed in this study during hexamer classification (Figure S1); examples of single HS-AFM frames used for SPA and the final classification step from which classes showing closed or open pores are obtained at pH 8 (Figure S2), pH 6 (Figure S3), and pH 8 + IP6 (Figure S5); comparison between experimental and simulated AFM images and topographic profiles (Figure S4); titration curves of control pentapeptides (Figure S6); cumulative and block-averaged p*K*_a_ in the absence of IP6 (Figure S7) and in the presence of IP6 (Figure S8); representative β-hairpin structures of clustering at different pH in the absence of IP6 (Figure S9) and in the presence of IP6 (Figure S10); populations of the clusters and HOLE radius profiles of the β-hairpin structures at different pH in the absence of IP6 (Figure S11) and in the presence of IP6 (Figure S12); class types of the pores from PAM clustering (Figure S13); classification models in the absence of IP6 (Figure S14) and in the presence of IP6 (Figure S15). Residue–residue distance distributions in the absence of IP6 (Figure S16) and in the presence of IP6 (Figure S17); description of measurements on mechanics, equilibrium dynamics, and fatigue resistance of the material (Figure S18); and example frames from Movie S1 and Movie S2 (PDF)
HS-AFM movie of the CA lattice at pH 8 (Movie S1) (AVI)
Movie showing the dynamics of a single hexamer (Movie S2) (AVI)

## ⊥

mAbxience (Genhelix), Julia Morros s/n, 24009 Armunia, León, Spain
