# Supplementary material for: Inositol Hexakisphosphate Promotes Open Pore State in HIV Capsid Protein Lattice by Reconfiguring Subunit Contacts
Source: ACS Nano. 2026 Sep 19;20(39):26496–510. doi: 10.1021/acsnano.6c07706 (PMC13648270; doi:10.1021/acsnano.6c07706)
Supplement: Supplementary file 1 [file nn6c07706_si_001.pdf]

SUPPLEMENTARY INFORMATION

Inositol hexakisphosphate promotes open pore state in HIV capsid protein lattice by reconfiguring subunit contacts

Yari Katar Knelissen,[1,¶] Judith Escrig,[2,¶] Amanda C. Macke[3], Sruthi Sudhakar[3], Santos Domínguez-Zotes,[2,+] Sourav Maity,[1] Mauricio G. Mateu,[2] Juan R. Perilla,[3,*] Wouter H. Roos[1,*] and Alejandro Valbuena[2,4,*]

[1]Moleculaire Biofysica, Zernike Instituut, Rijksuniversiteit Groningen, 9747 AG, Groningen, The Netherlands

[2]Centro de Biología Molecular Severo Ochoa (CSIC-UAM), Campus of the Universidad Autónoma de Madrid, 28049 Madrid, Spain

[3]Department of Chemistry and Biochemistry, University of Delaware, Newark, DE 19716 USA

[4]Departamento de Biología Molecular, Universidad Autónoma de Madrid, 28049 Madrid, Spain

[¶] These authors contributed equally to this work

[+]Present address: mAbxience (Genhelix), Julia Morros s/n, 24009 Armunia, León, Spain

*Correspondence to A.V.: alejandro.valbuena@uam.es, avalbuena@cbm.csic.es; ORCID: 0000-0002-6801-640X; and W.H.R.: w.h.roos@rug.nl; or J.R.P.: jperilla@udel.edu.

Keywords: high speed AFM, human immunodeficiency virus, capsid protein lattice, pore opening, molecular dynamics, constant-pH molecular dynamics

The CA hexamer free energy landscape is modulated by IP6, leading to pore opening

The data obtained by using SPA uniquely allowed the quantification of the proportion of individual hexamers in either the open or close pore conformation. From these data the free energy difference between the two conformations was quantified under different conditions, including different pHs and the absence or presence of IP6. To this end it was considered that each state is populated according to the Boltzmann distribution, assuming a strict two-state equilibrium and negligible perturbation of populations by the lattice environment and AFM imaging process. Results of the free energy calculations are shown in Table 1. The free energy difference between C and O states at pH 8 is slightly positive ($\Delta G_{C\text{-}O}^{pH8}$=0.34±0.18 kcal/mol), indicating that at this pH the closed state is slightly more stable than the open state. In contrast, a mild acidification to pH 6 led to a significant stabilization of the open state relative to the closed state ($\Delta G_{C\text{-}O}^{pH6}$=-2.67± 0.19 kcal/mol). Likewise, addition of IP6 at pH 8 led to a significant stabilization of the open state relative to the closed state ($\Delta G_{C\text{-}O}^{pH8+IP6}$=-0.84±0.12 kcal/mol). In summary, the difference in free energy between the closed and the open pore states is marginal under the conditions tested; both a mild acidification and binding of IP6 to the CA protein lattice promote the opening of the capsid pores. The difference in free energy between the open state (O) and closed state (C) in different conditions was calculated taking into account that the pores are close to equilibrium and that the number of states observed is exponentially related to their energy through the Boltzmann distribution. Specifically, considering the canonical theory of the transition state of the reaction rate, the effective free energy difference between closed and open pores ($\Delta G_0^{C\text{-}O}$) was determined as follows:

$$\Delta G_0^{C-O} = -k_b T \cdot ln\left(\frac{N_O}{N_C}\right) \qquad \text{(S1)}$$

where N_O is the number of open pores, N_C is the number of closed pores, k_b is the Boltzmann constant and T is the temperature.

Table S1. Number of frames and particles analyzed by two independent users during the classification workflow. From top to bottom, the number of frames, particles, classes, and particles assigned to open or closed states are specified for the three conditions used. In the case of pH8+IP6, it was necessary to perform additional rounds of classification to eliminate incomplete hexamers with poor resolution.

	Condition	pH8		pH6	
	User	1	2	1	2
Round 1	Frames	250		273	
	Particles	4162	3262	4921	2652
	Classes	8		8	
	Good	2414	1552	2136	956
Round 2	Classes	6		6	24
	Open	992	448	1992	793
	Close	1422	824	27	7

	Condition	pH8+IP6	
	User	1	2
Round 1	Frames	1436	
	Particles	20626	17395
	Classes	12	
	Good	11231	7244
Round 2	Classes	24	
	Good	7424	4323
Round 3	Classes	6	
	Open	5998	3305
	Close	1269	926

Table S2. Comparison of pK_a values obtained from the CpHMD simulations of the CA hexamers in fixed and varying IP6 formal charge states. pK_a values are reported per protomer chain (A-F) from 10,000 cycles after dropping the first 2,000 cyles as the mean ± standard deviation across all six chains. The variable-charge model assigns IP6 charge of −6 at low pH, −8 at mid pH and −10 at high pH.

	H12		E45	
**IP6 Charge state**	**−12**	**−6/−8/−10**	**−12**	**−6/−8/−10**
**Chain A**	7.5	6.2	6.4	7.2
**Chain B**	6.4	6.6	6.6	6.1
**Chain C**	5.1	6.7	6.9	6.2
**Chain D**	7.9	6.8	5.6	5.9
**Chain E**	5.9	6.5	7.0	6.3
**Chain F**	6.6	6.3	5.8	6.5
**Average**	**6.5±1.0**	**6.3±0.5**	**6.4±0.6**	**6.4±0.4**

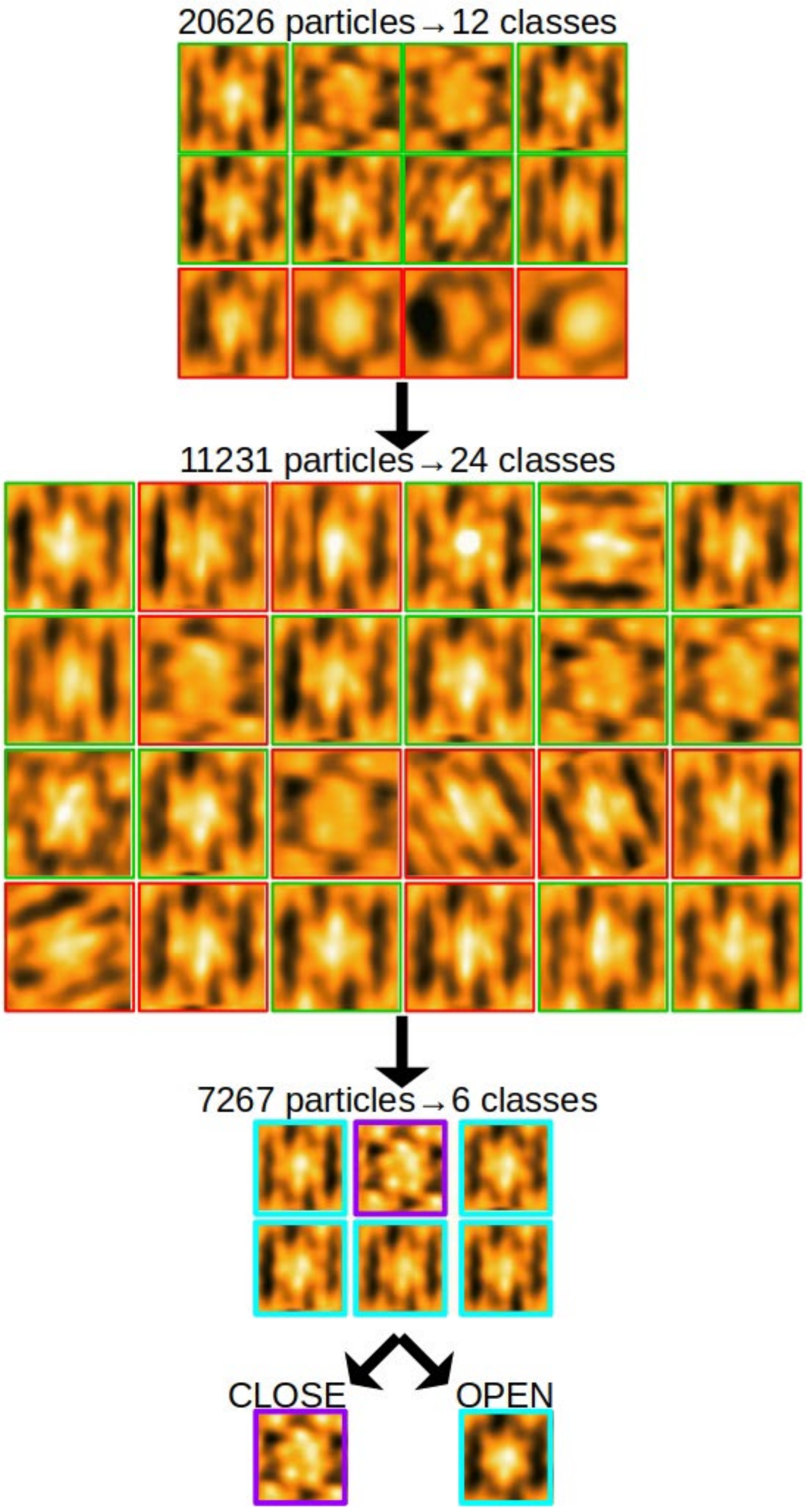

Figure S1. Example workflow for removing and classifying CA hexamers showing the pore in the open or closed state at pH 8 in the presence of IP6. The automatically extracted particles (hexamers) are entered as input for the first classification round into 12 classes. The particle average of the classes included in the subsequent round of classification are indicated with green squares, while the eliminated classes are indicated with red squares. After the second classification round into 24 classes, all classes indicated in green were used for the classification round into 6 classes. Each of the classes showing the bulge (cyan squares) or a flat center (purple squares) were used to obtain the consensus of the hexamer with the bulge or flat (Open or Close, respectively). The number of hexamers in each round is indicated and corresponds to those shown in Table S1. Analysis performed by user 2.

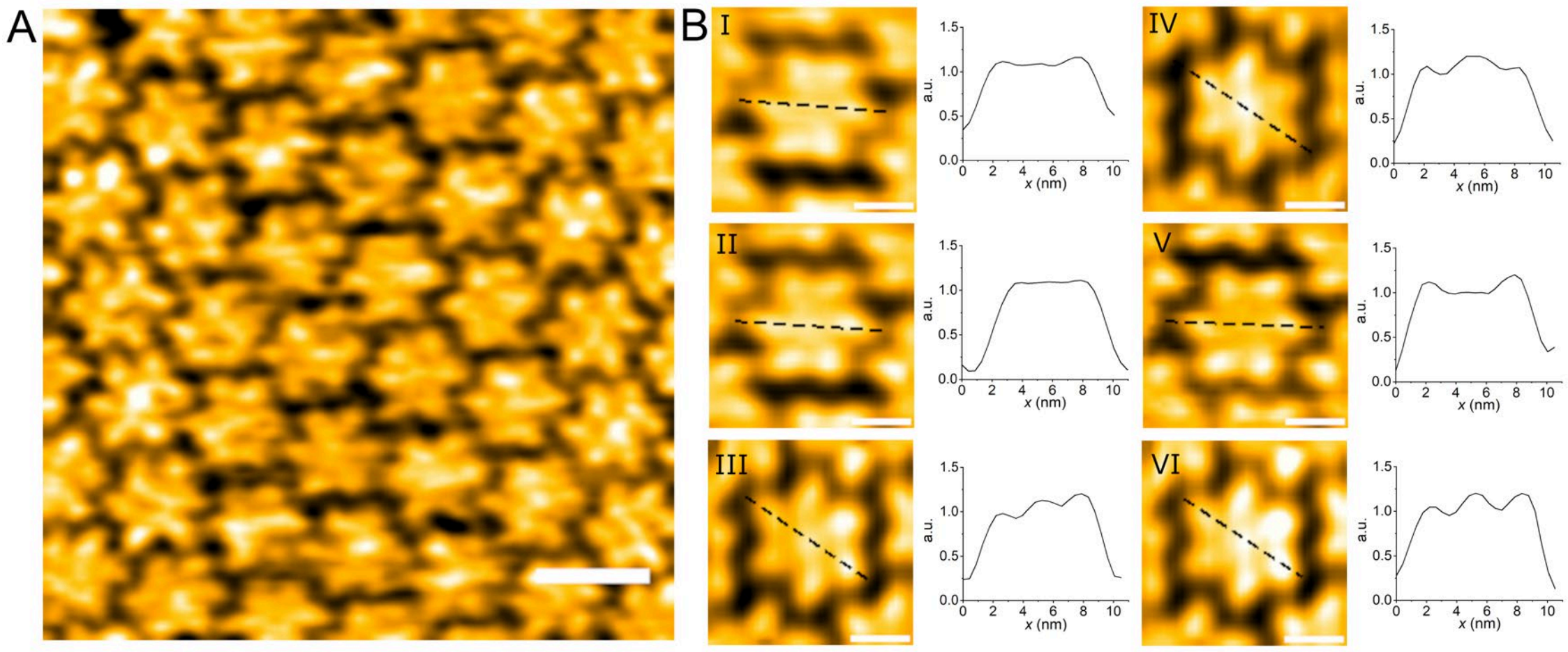

Figure S2. Single particle analysis of CA hexamers at pH 8. A) single HS-AFM frame showing CA hexamers. Scale bar corresponds to 10 nm. **B)** class-averaged hexamers from I to VI. Scale bars correspond to 5 nm. Profile lines along the dashed black lines on the averaged images are on the right side of each class average. Analysis performed by user 1.

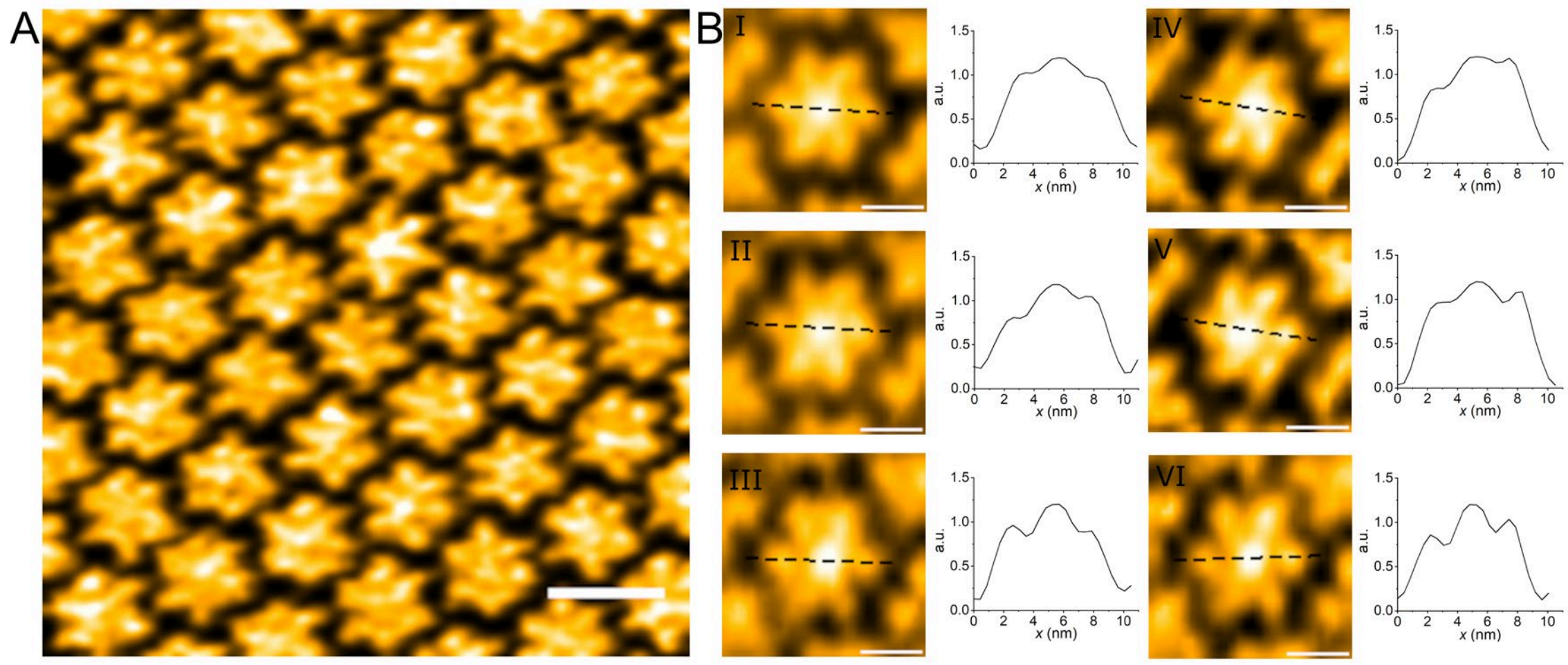

Figure S3. Single particle analysis of CA hexamers at pH 6. A) single HS-AFM frame showing CA hexamers. Scale bar corresponds to 10 nm. **B)** class-averaged hexamers from I to VI. Scale bars correspond to 5 nm. Profile lines along the dashed black lines on the averaged images are on the right side of each class average. Analysis performed by user 1.

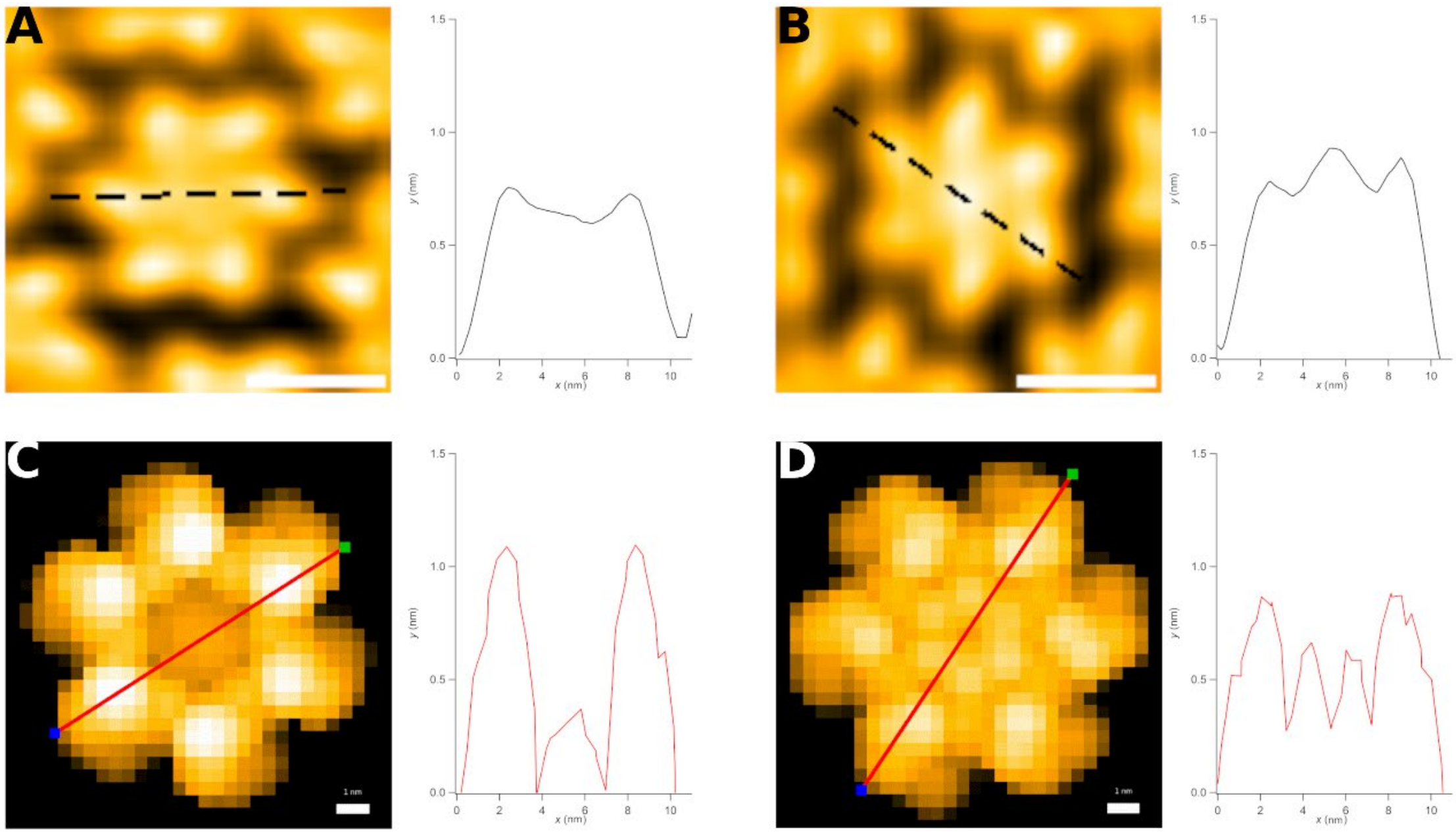

Figure S4. Comparison of CA hexamer topography visualized by HS-AFM and simulated from atomic structure. A) averaged HS-AFM image of the closed pore. Black line indicates the profile height shown on the right side. Scale bar corresponds to 5 nm. **B)** averaged HS-AFM image of the open pore. Black line indicates the profile height shown on the right side. Scale bar corresponds to 5 nm. **C)** simulated AFM image from atomic structure of the closed state (PDB:6B2J). Red line indicates the profile height shown on the right side. Scale bar corresponds to 1 nm. **D)** simulated AFM image from atomic structure of the open state (PDB:6B2I). Red line indicates the profile height shown on the right side. Scale bar corresponds to 1 nm.

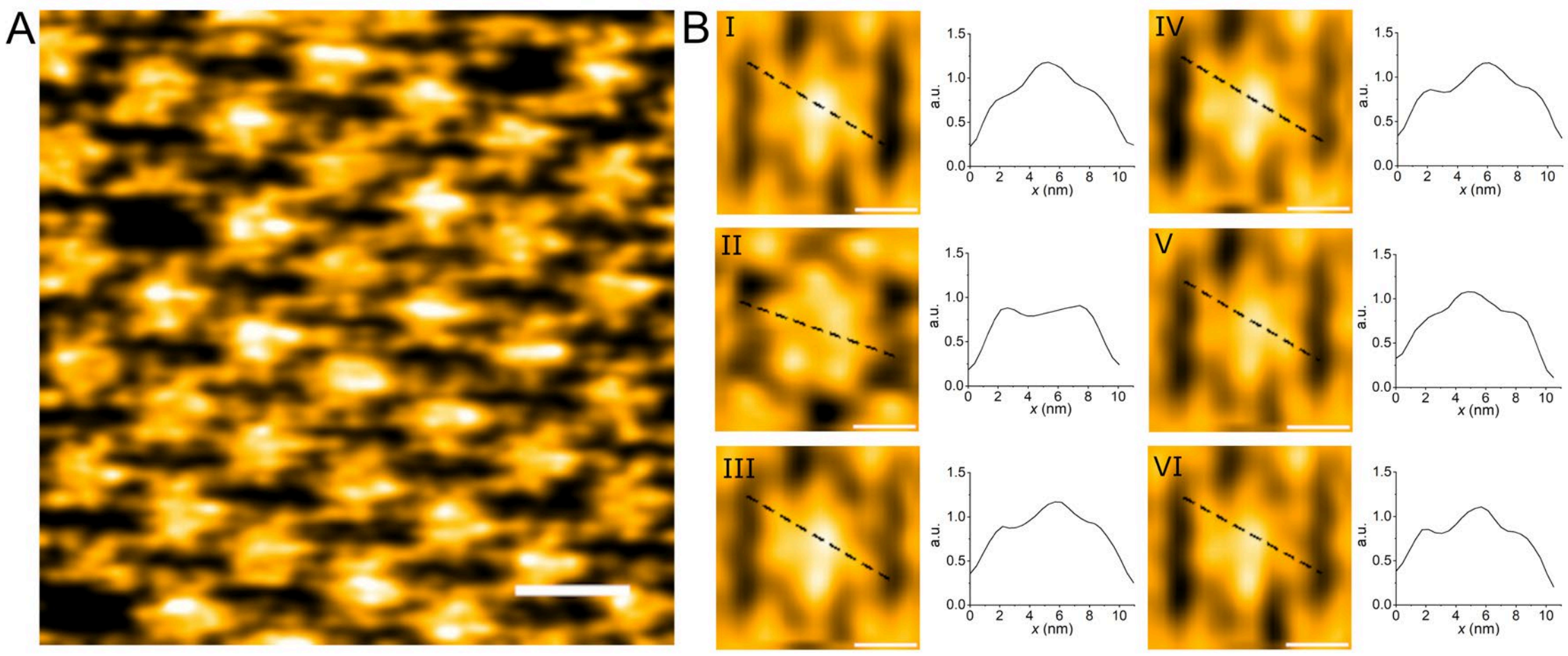

Figure S5. Single particle analysis of CA hexamers at pH 8 in the presence of IP6. A) single HS-AFM frame showing CA hexamers. Scale bar corresponds to 10 nm. **B)** class-averaged hexamers from I to VI. Scale bars correspond to 5 nm. Profile lines along the dashed black lines on the averaged images are on the right side of each class average. Analysis performed by user 1.

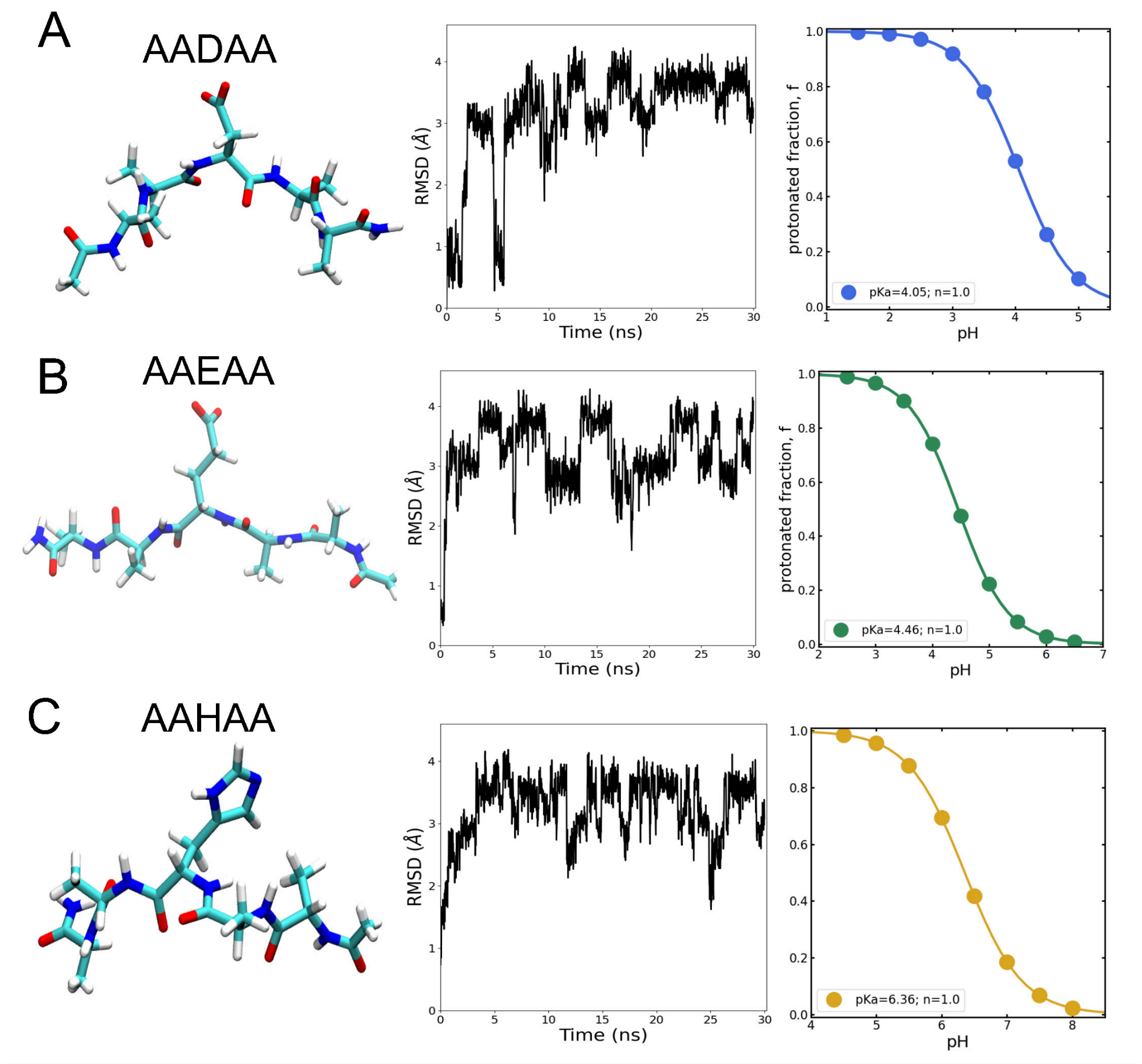

Figure S6. Pentapeptide MD RMSD and CpHMD titration curves. Representative final structure, RMSD profile from 30 ns MD equilibration simulations, and titration curve from CpHMD assay of the model pentapeptides: **A)** Asp (pH range: 1.5 to 5.0), **B)** Glu (pH range: 2.5 to 6.5), and **C)** His (pH range: 4.5 to 8.0). Calculated pK_a values are indicated. All structures are visualized with VMD.

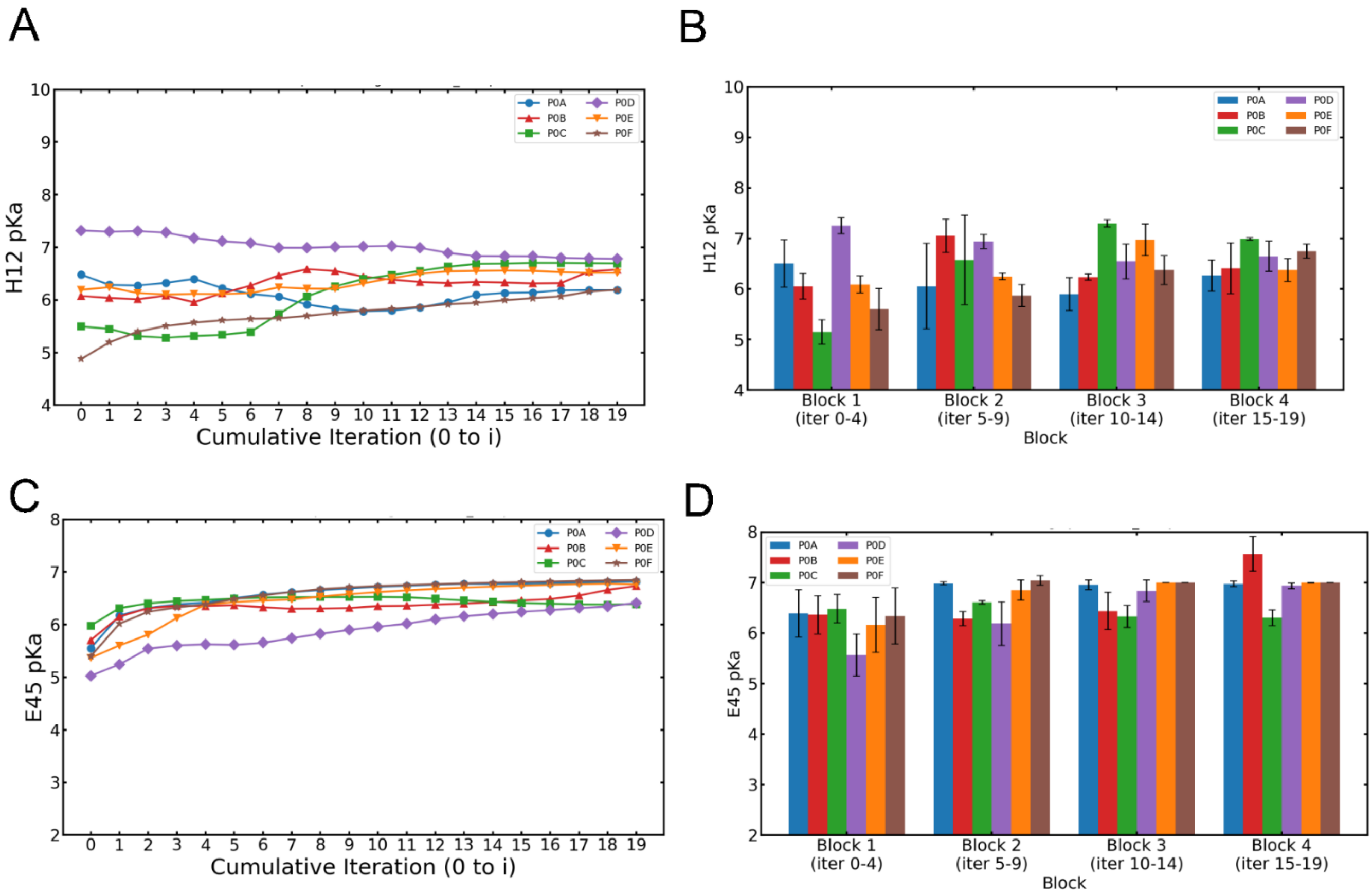

Figure S7. Cumulative and block-averaged pK_a convergence for H12 and E45 in the absence of IP6. pK_a convergence was assessed using cumulative pooled sampling windows (iterations 0 through *i*, corresponding to 2,000-40,000 neMD/MC cycles per pH condition) and four independent, non-overlapping trajectory blocks (iterations 0-4, 5-9, 10-14, 15-19), computed separately for each of the six CA chains. (A) and (B) are H12; (C) and (D) are E45. (A) and (C) show cumulative pK_a convergence; (B) and (D) show block-averaged pK_a with standard deviation across the four blocks.

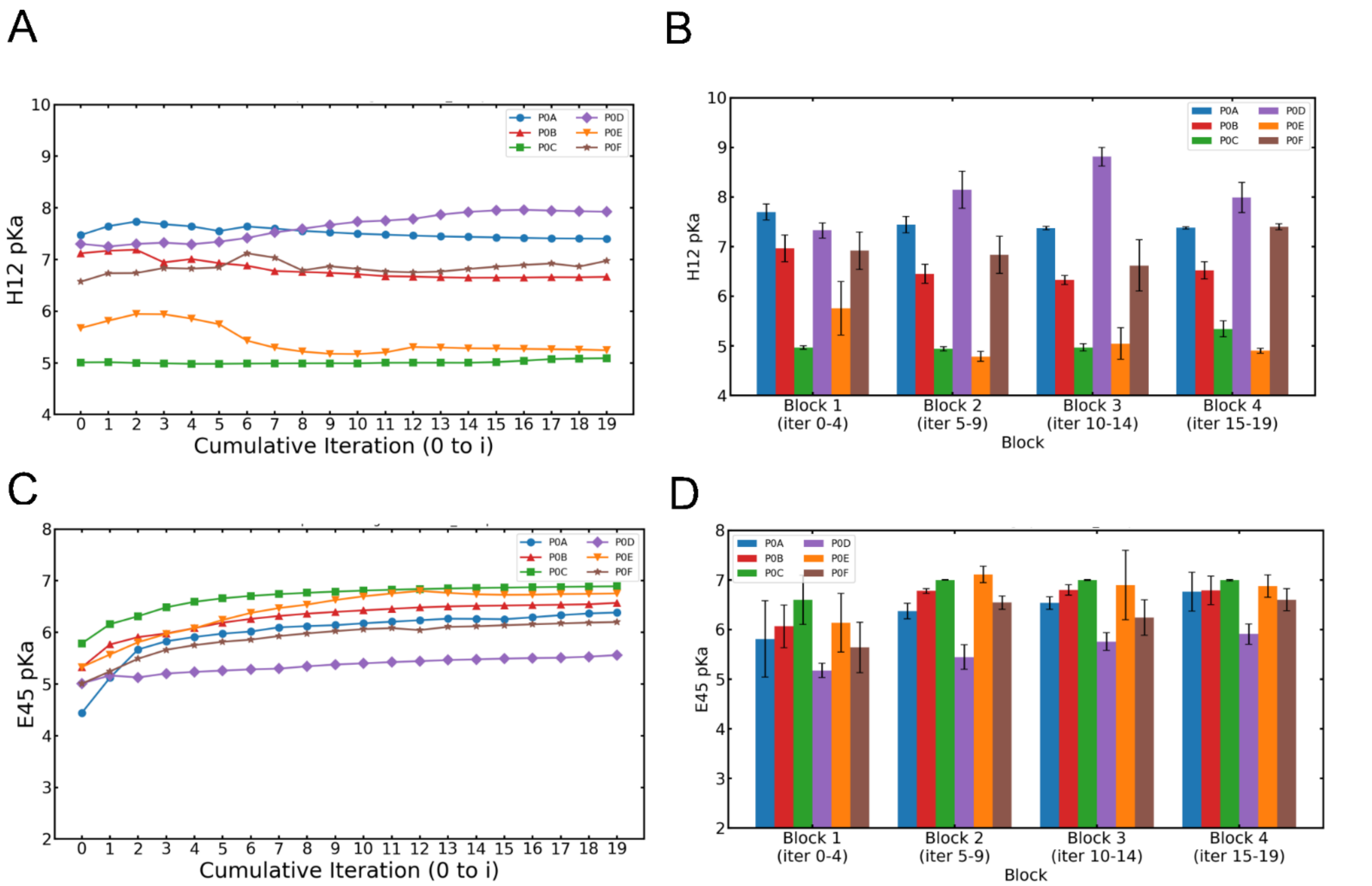

Figure S8. Cumulative and block-averaged pK_a convergence for H12 and E45 in the presence of IP6. pK_a convergence was assessed using cumulative pooled sampling windows (iterations 0 through *i*, corresponding to 2,000-40,000 neMD/MC cycles per pH condition) and four independent, non-overlapping trajectory blocks (iterations 0-4, 5-9, 10-14, 15-19), computed separately for each of the six CA chains. (A) and (B) are H12; (C) and (D) are E45. (A) and (C) show cumulative pK_a convergence; (B) and (D) show block-averaged pK_a with standard deviation across the four blocks.

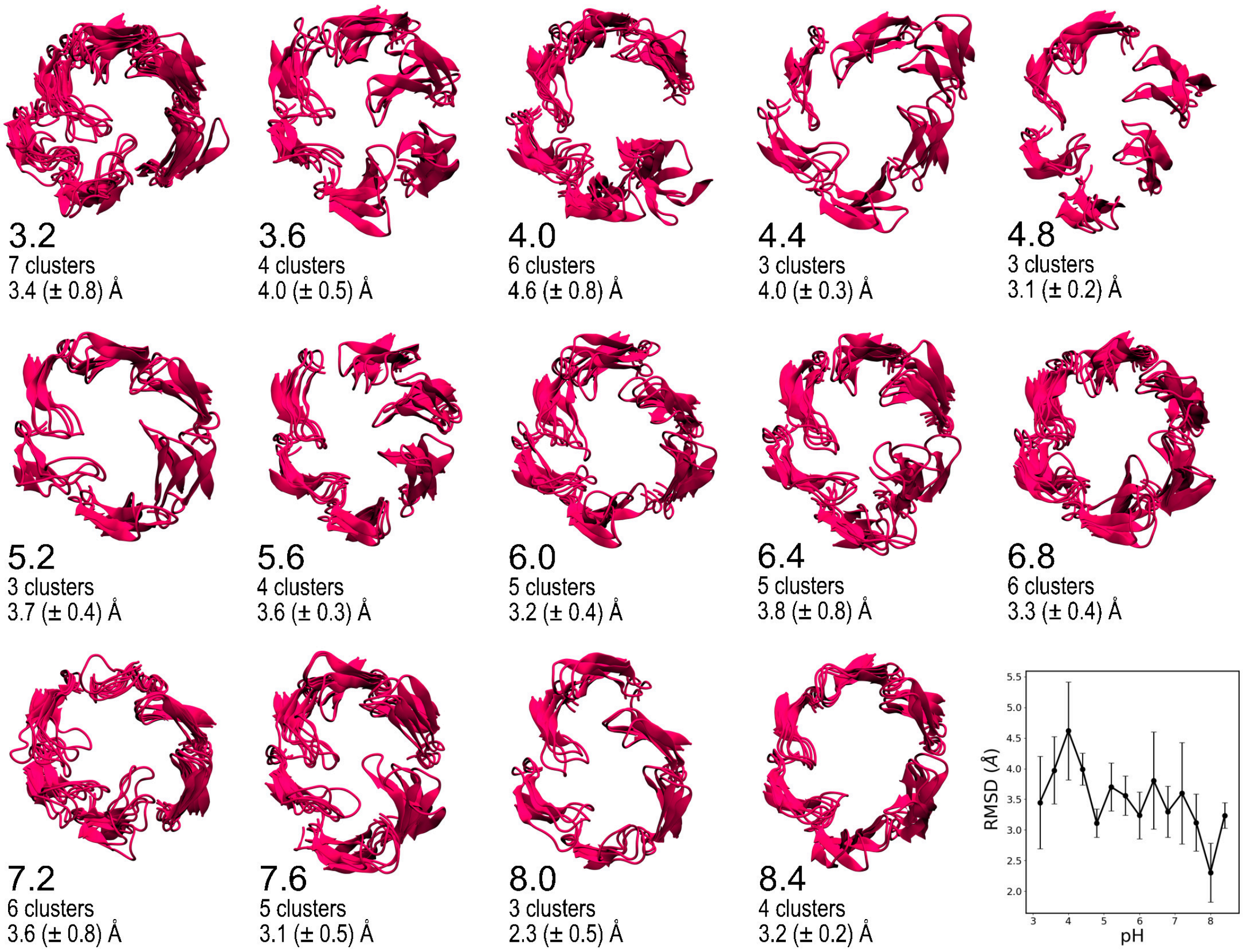

Figure S9. All cluster representative structures of the β-hairpin within CA hexamer across the sampled pH in the absence of IP6. Cluster representative structures of the central pore in the absence of IP6 across the sampled pH using the PAM clustering algorithm. The pH, the number of clusters and the RMSD (± standard deviation) are indicated in the left-hand corner of each β-hairpin structure. In the bottom right-hand corner, the average RMSD (± standard deviation) of all conformations at each pH relative to the first cluster structure, with error bars indicating conformational variability within each ensemble.

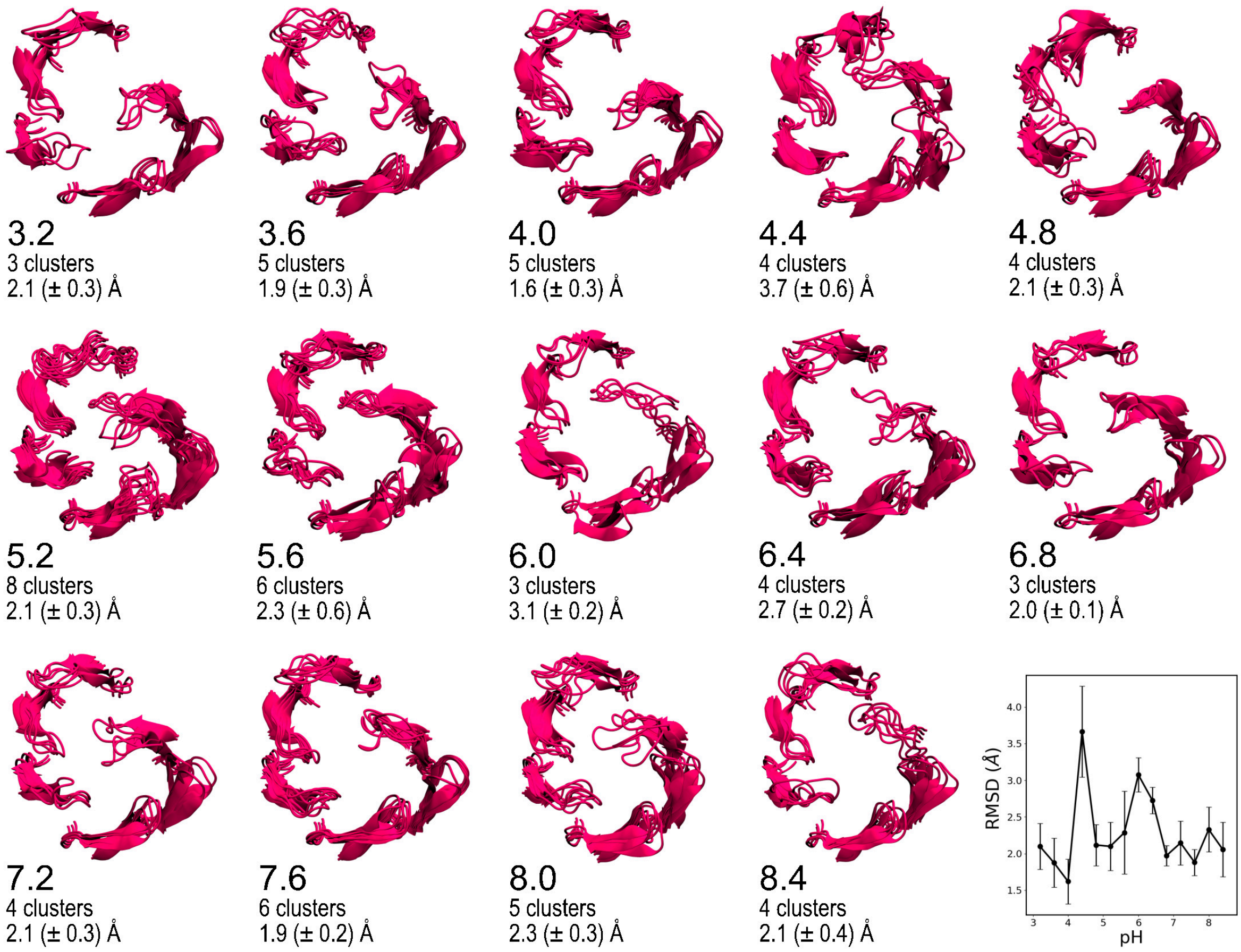

Figure S10. All cluster representative structures of the β-hairpin within CA hexamer across the sampled pH in the presence of IP6. Cluster representative structures of the central pore in the presence of IP6 across the sampled pH using the PAM clustering algorithm. The pH, the number of clusters and RMSD (± standard deviation) are indicated in the left-hand corner of each β-hairpin structures. In the bottom right-hand corner, the average RMSD (± standard deviation) of all conformations at each pH relative to the first cluster structure, with error bars indicating conformational variability within each ensemble.

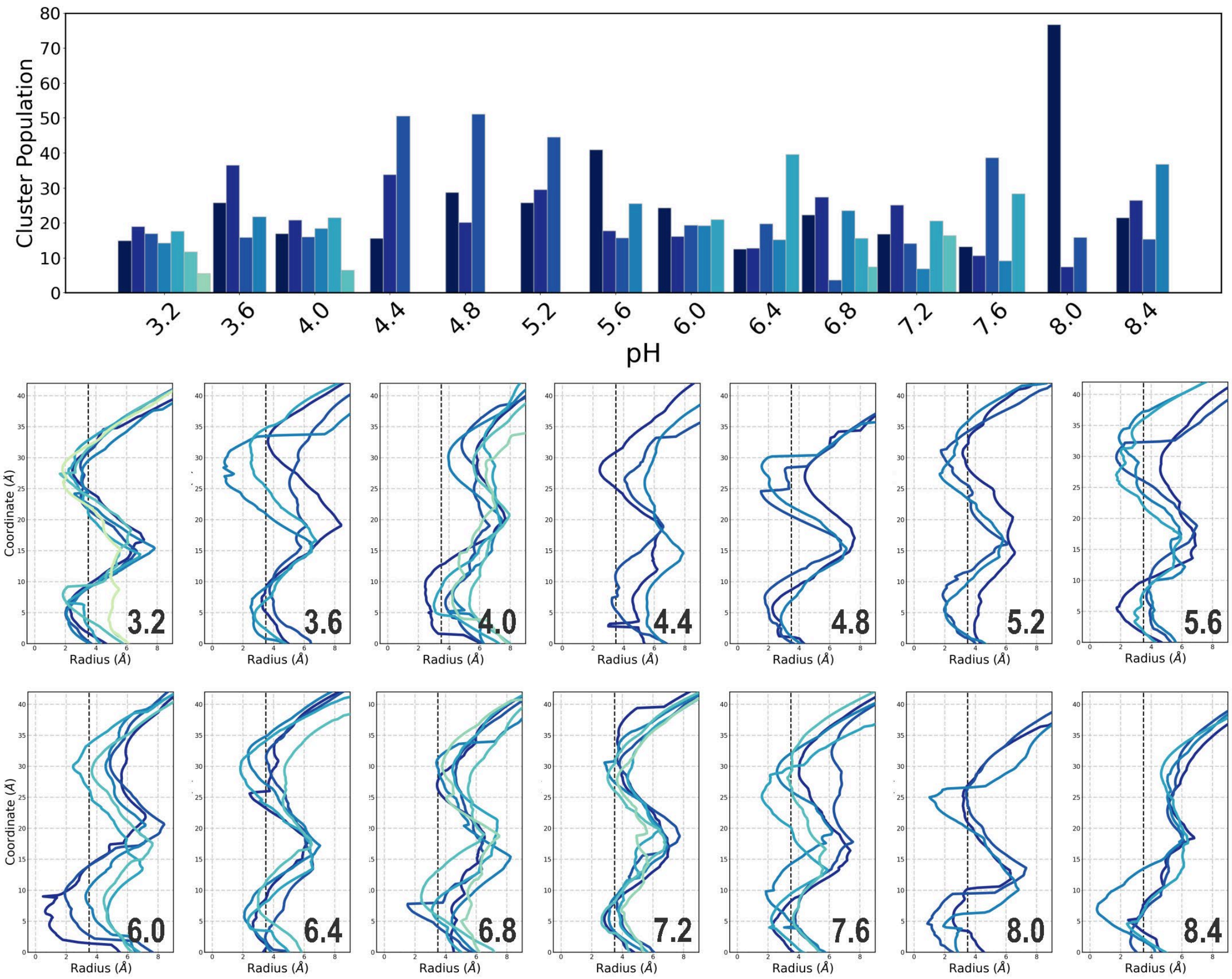

Figure S11. Cluster populations and HOLE radius profiles of the hexameric β-hairpin assembly in the absence of IP6. In the top panel, a bar plot of the cluster populations is provided across each sampled pH. On the bottom, the channel pore radius from the HOLE2 analysis of each cluster representative structure. A dotted line is provided at a radius of 3.5 Å which would correspond to the approximate length of a dNTP (~6-7Å) which suggests a potential limitation to the transportation of dNTPs. This analysis was used to assign the appropriate class in the XGBoost classification model.

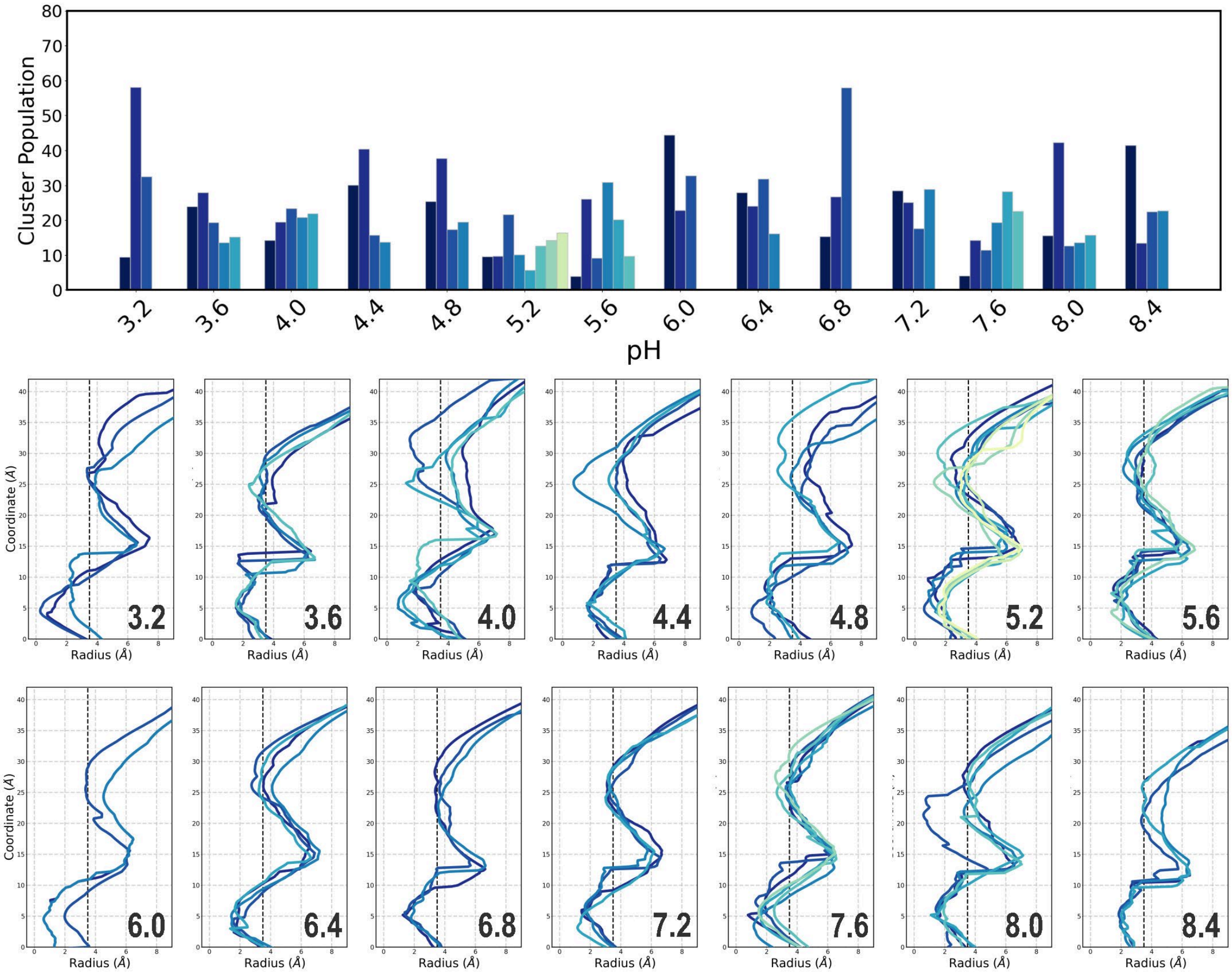

Figure S12. Cluster populations and HOLE radius profiles of the hexameric β-hairpin assembly in the presence of IP6. In the top panel, a bar plot of the cluster populations is provided across each sampled pH. On the bottom, the channel pore radius from the HOLE2 analysis of each cluster representative structure. A dotted line is provided at a radius of 3.5 Å which would correspond to the approximate length of a dNTP (~6-7Å) which suggests a potential limitation to the transportation of dNTPs. This analysis was used to assign the appropriate class in the XGBoost classification model.

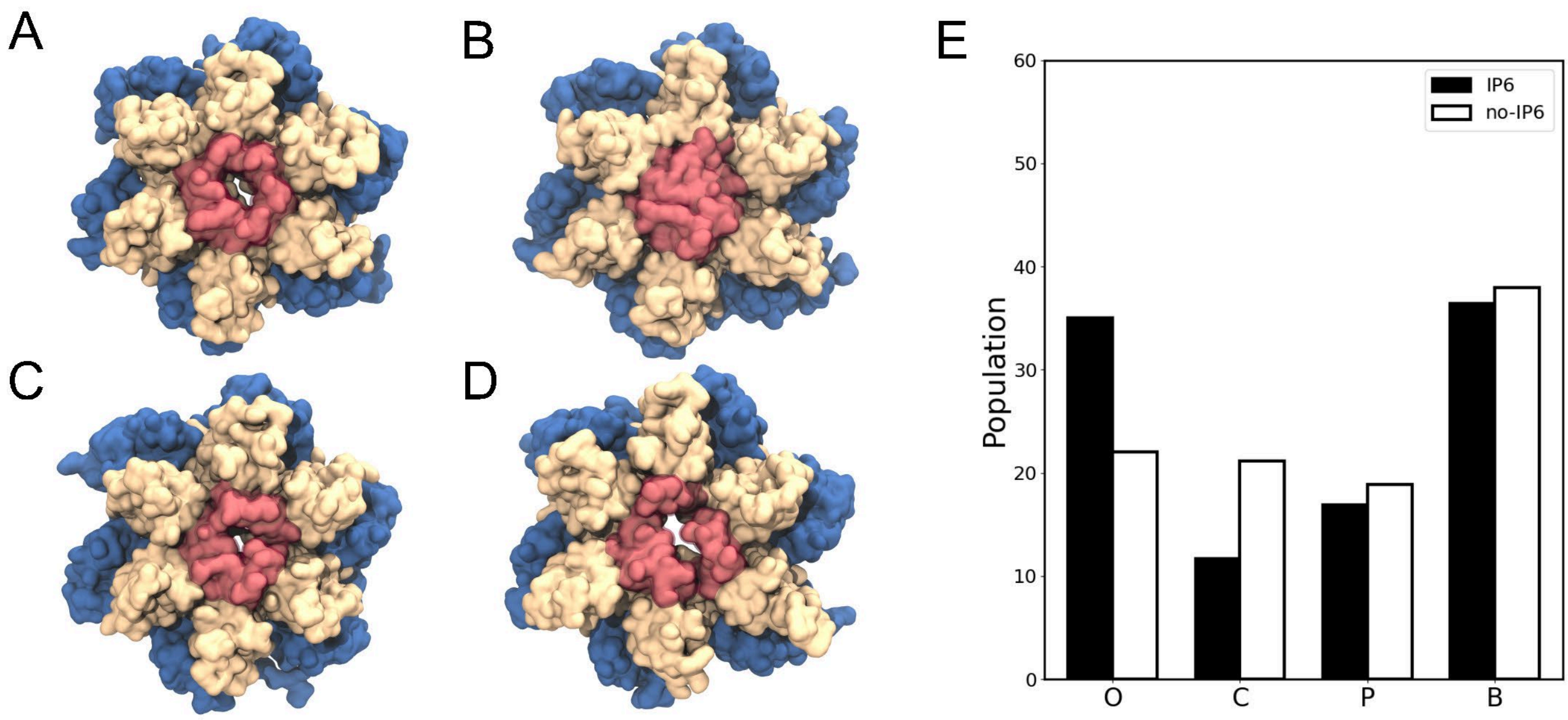

Figure S13. Characterized class types from PAM cluster representative structures. Snapshots of HOLE2 pathways for **A)** "Open" **B)** "Closed", **C)** "Protruding", and **D)** "Broken". **E)** The populations of each class observed in the ensemble in the presence of IP6 (black) and the absence of IP6 (white).

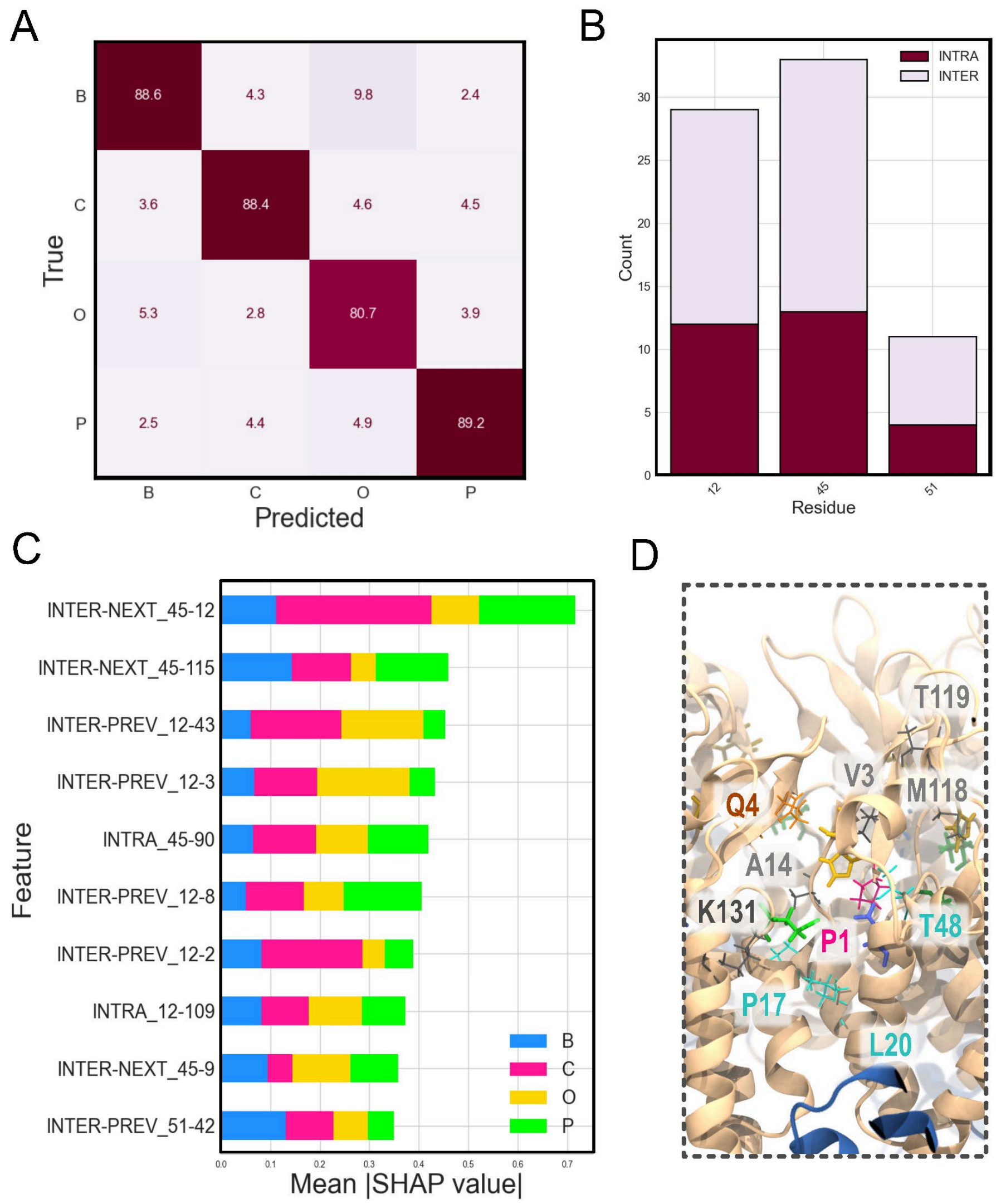

Figure S14. Classification model summary in the absence of IP6. A) the resulting confusion matrix for the selected model built from the top 70 features. **B)** the breakdown of the type of pairwise distance by titratable group and intra- *vs* inter- protomer. This demonstrates a minimal contribution of D51 to pore dynamics whereas the H12 and E45 contribution is comparable. **C)** mean SHAP values for the top 10 features, highlighting the contribution of each pairwise distance to the prediction of each class. **D)** the residues found to participate in pH-dependent contacts with the titratable groups. H12 is yellow, E45 is green, and D51 is in blue. The domains are colored such that the NTD is tan and the CTD is navy, and the residues are colored such that intra-protomer residues are in gray, $H12_{i\text{ - }i\text{-}1}$ are orange, $E45_{i\text{ - }i\text{+}1}$ are cyan, and contacts that form with H12 and D51 are in magenta.

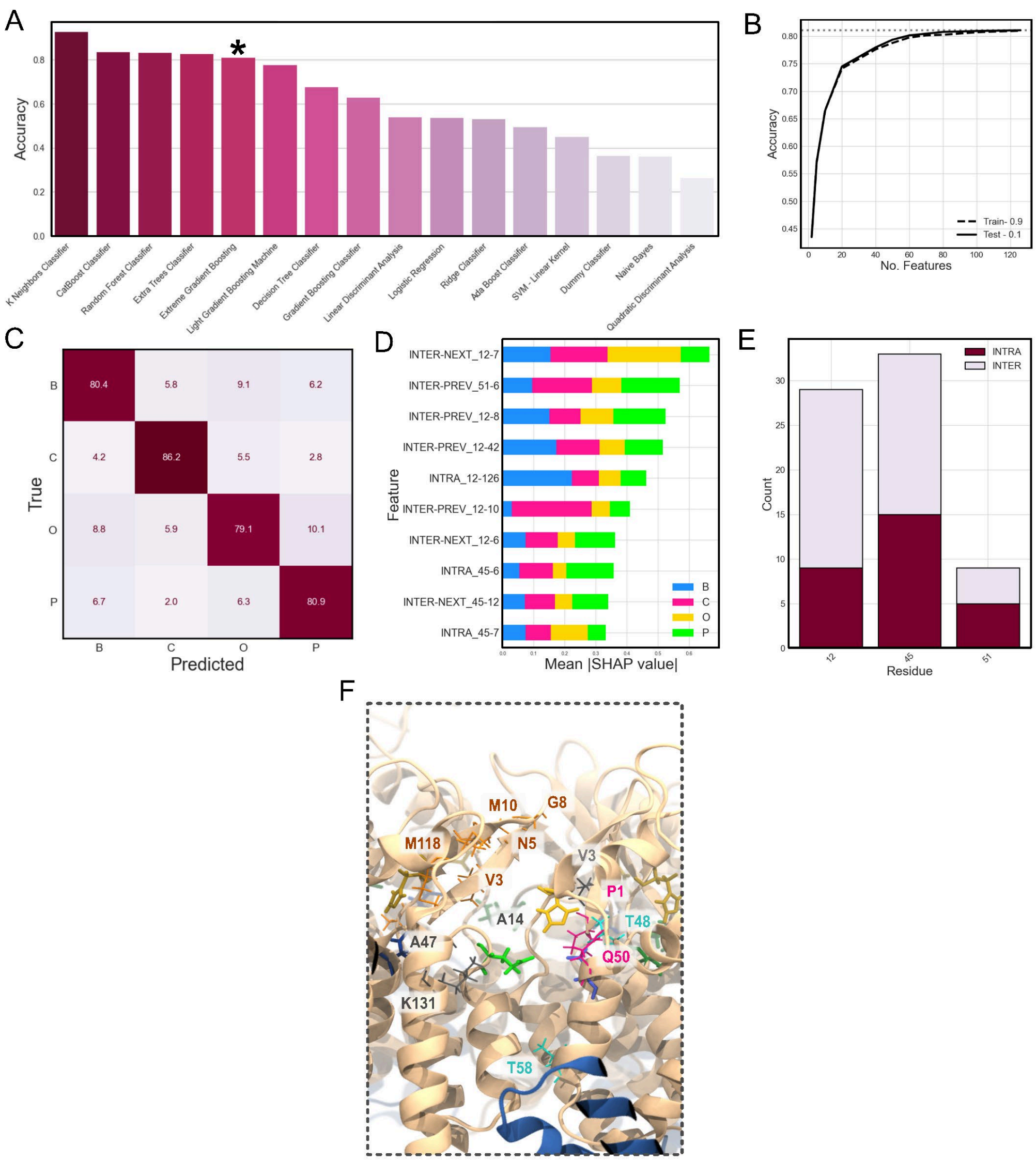

Figure S15. Classification model summary in the presence of IP6. A) summary of classification accuracy across the evaluated ensemble of algorithms, used to evaluate a suitable model for the dataset. XGBoost evaluate was demonstrated as one of the top-performing classifiers (*), and was selected for the numerical feature space. **B)** accuracy scores for the training and test sets as the number of features was progressively reduced, allowing identification of the minimal feature set required to maintain optimal performance. **C)** the resulting confusion matrix for the selected model. **D)** mean SHAP values for the top 10 features, highlighting the contribution of each pairwise distance to the prediction of each class. **E)** the breakdown of the type of pairwise distance by titratable group and intra- *vs* inter- protomer. This demonstrates that D51 has a minimal contribution to pore dynamics whereas the H12 and E45 contribution is comparable. **F)** The residues found to participate in pH-dependent contacts with the titratable groups. H12 is yellow, E45 is green, and D51 is in blue. The domains are colored such that the NTD is tan and the CTD is navy, and the residues are colored such that intra-protomer residues are in gray, $H12_{i-i-1}$ are orange, $E45_{i-i+1}$ are cyan, and contacts that form with H12 and E45 are in magenta.

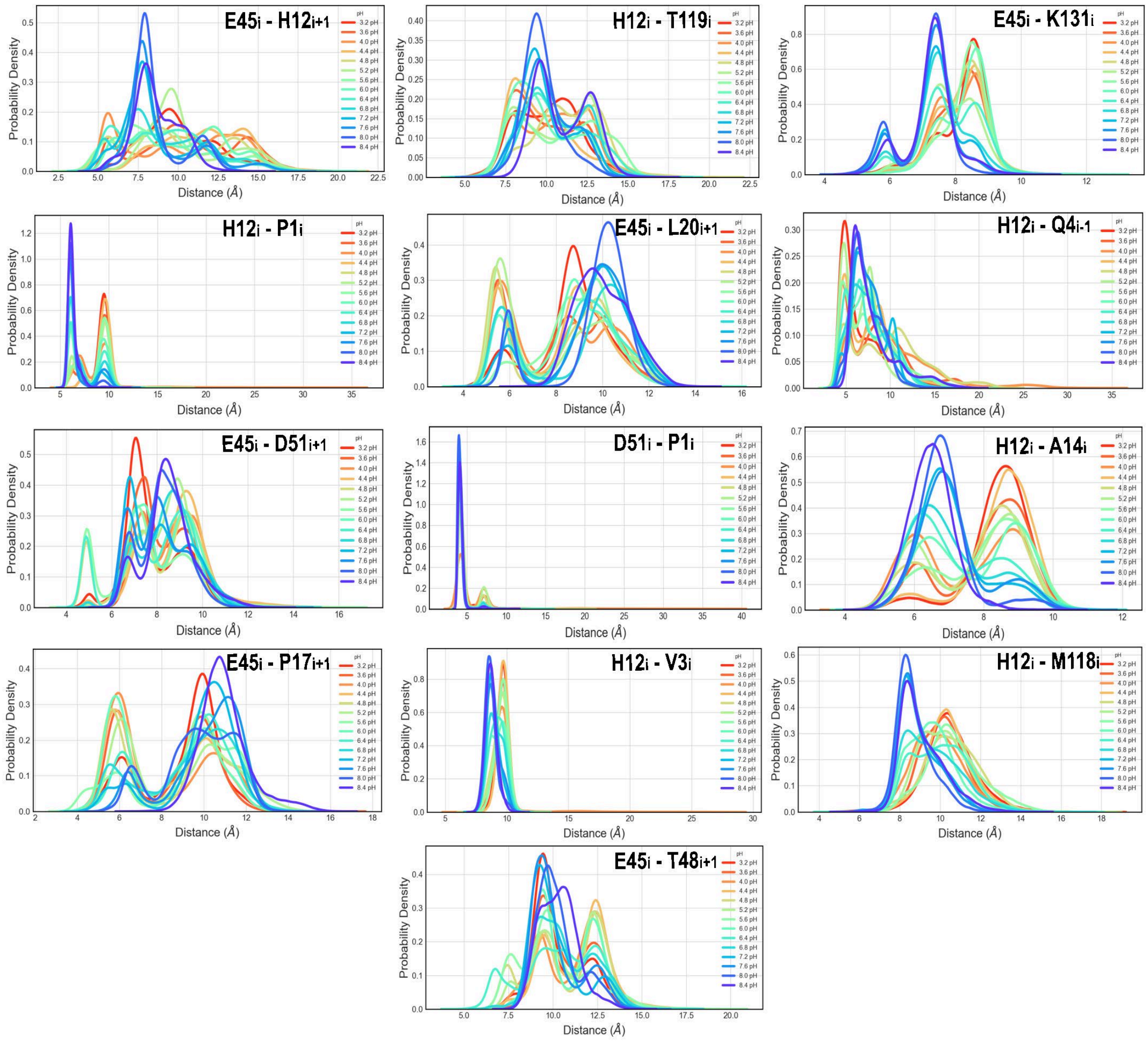

Figure S16. Distance distributions between residues in the absence of IP6. Distributions of the distance between residues contributing to the pore state in the analyzed pH range. The distribution corresponding to a specific pH is color-coded as shown in the box on the right. The specific residue pair is indicated at the top of the graph, with a subscript (i, i+1, or i-1) indicating whether the residue pair corresponds to an intra-chain (i, i) or inter-chain residue pair (i, i+1 or i, i-1).

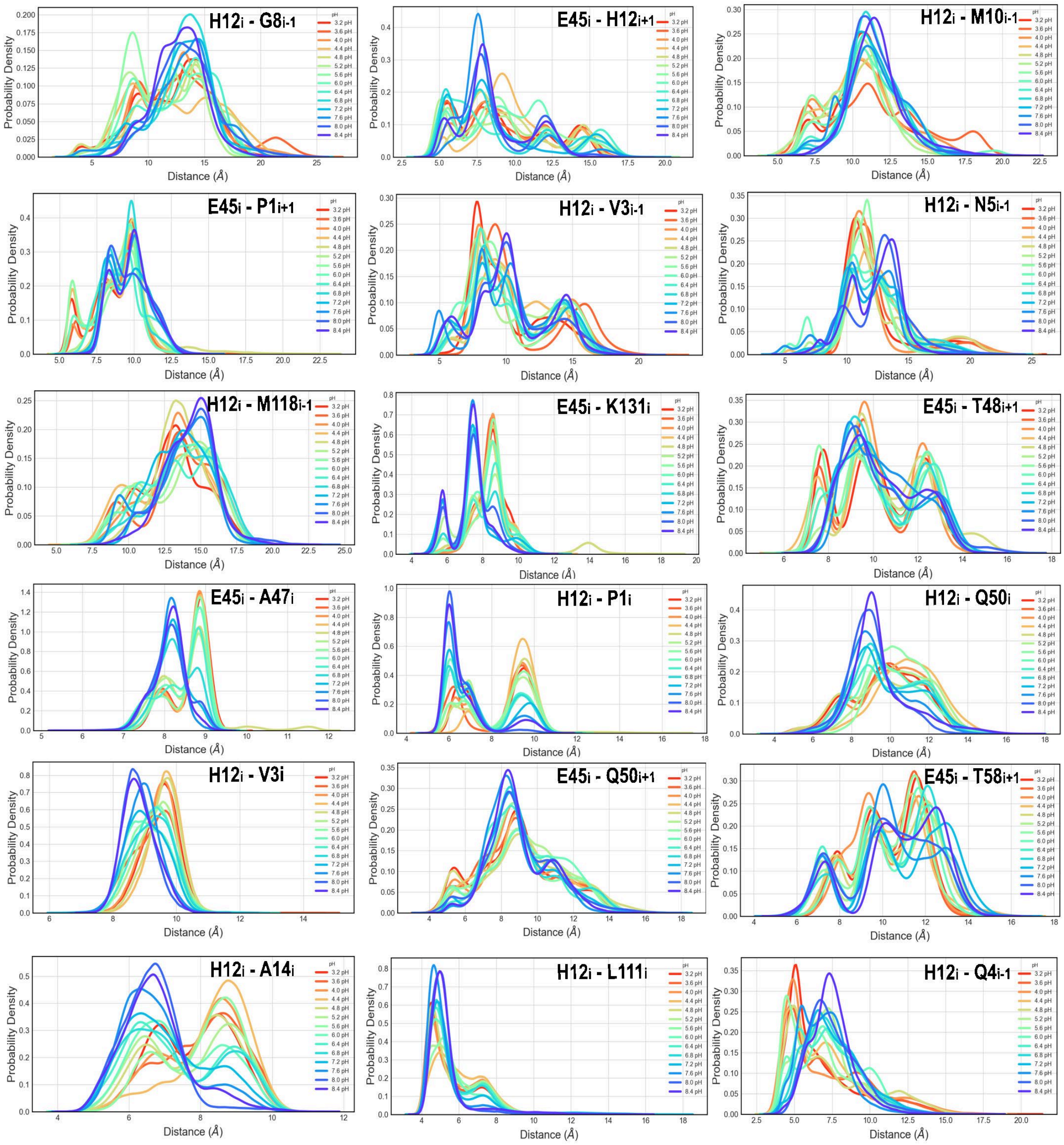

Figure S17. Distance distributions between residues in the presence of IP6. Distributions of the distance between residues contributing to the pore state in the analyzed pH range. The distribution corresponding to a specific pH is color-coded as shown in the box on the right. The specific residue pair is indicated at the top of the graph, with a subscript (i, i+1, or i-1) indicating whether the residue pair corresponds to an intra-chain (i, i) or inter-chain residue pair (i, i+1 or i, i-1).

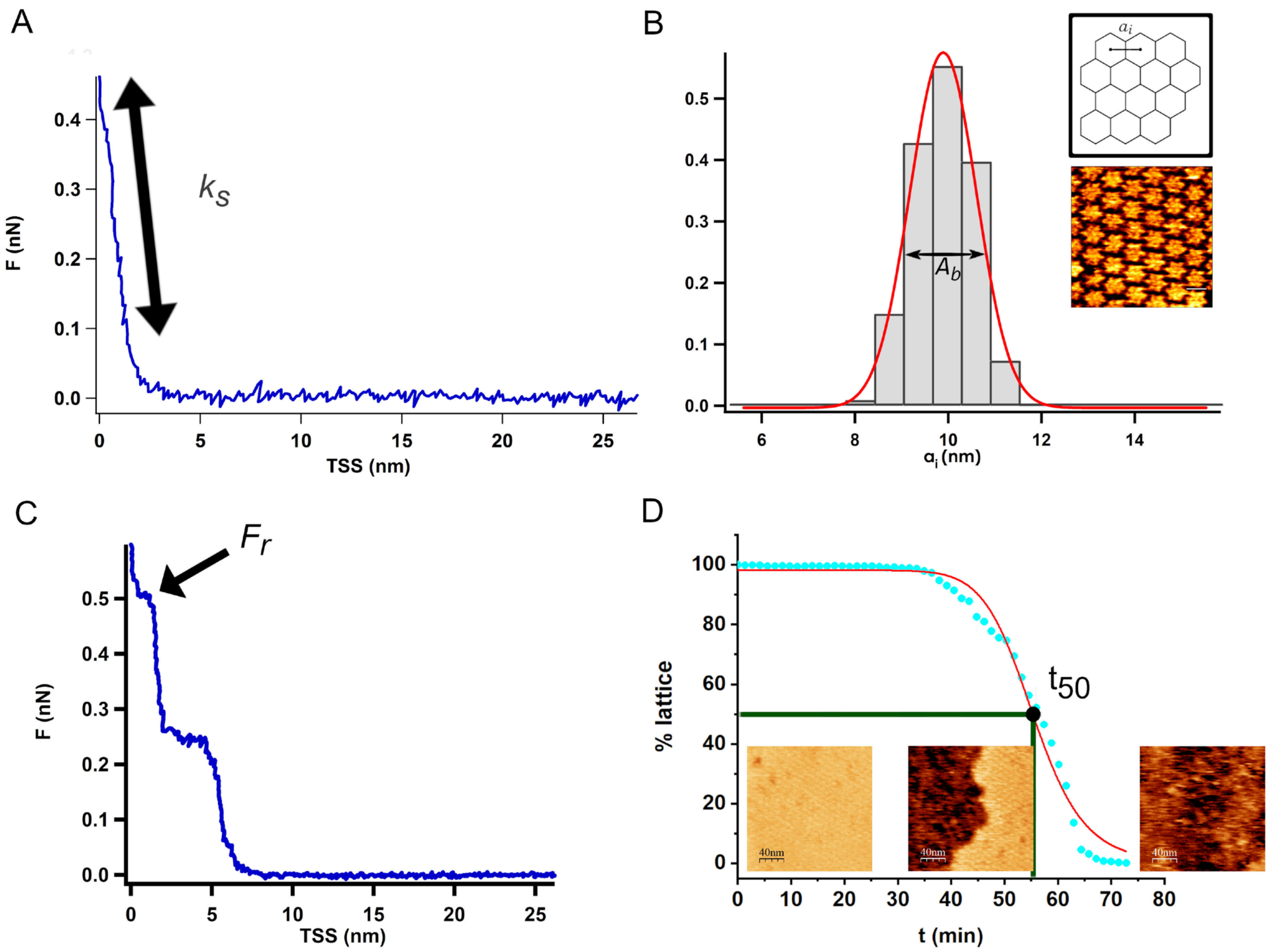

Figure S18. AFM-based measurements of CA lattice mechanics, stability and equilibrium dynamics. A) example of a Force *vs* Tip Sample Separation curve used to determine the spring constant (k_s). It is indicated, by double headed arrow, the range where the spring constant is measured. **B)** example of a hexamer-to-hexamer distances (a_i) distribution. The amplitude of breathing (A_b) is taken as the width of the Gaussian fitting (red curve). A scheme of the hexameric CA lattice is shown in the inset. **C)** example of a Force *vs* Tip Sample Separation curve used to determine the rupture force (F_r). The rupture force is taken as the force where the last rupture event occurs. **D)** initially, a region completely covered by the CA lattice (left inset) is mechanically fatigued until the CA lattice is completely removed (right inset). The time t_{50} is the time at which the area covered by the CA network reaches 50% of the initially scanned area (central inset).

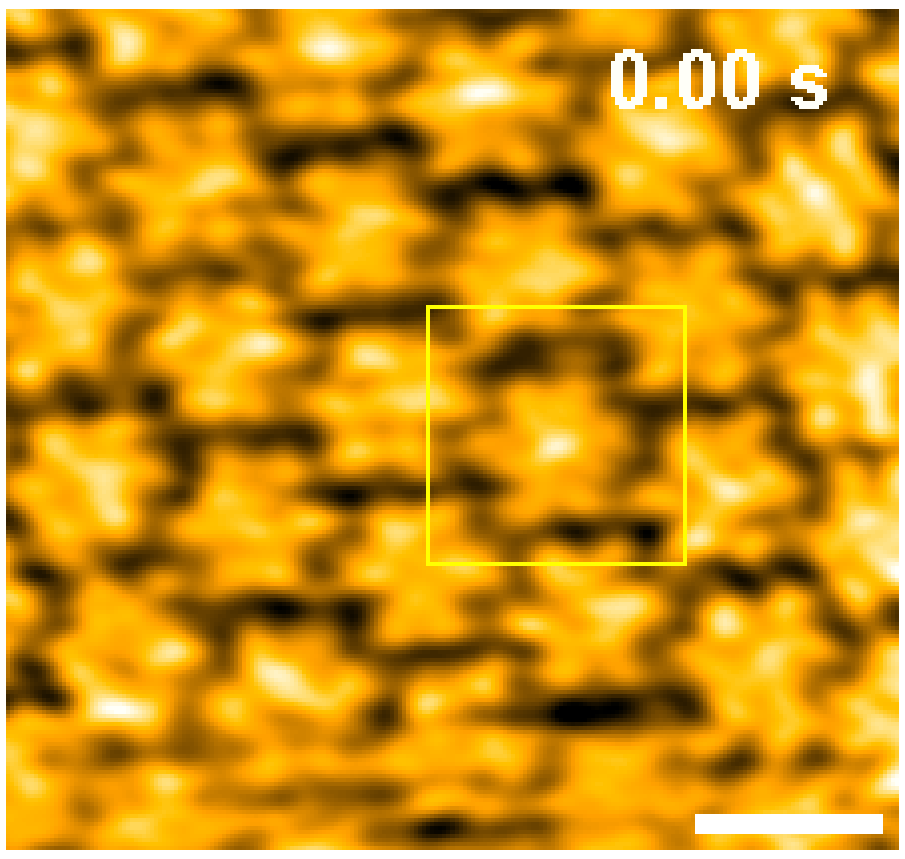

Movie S1. Frame extracted from HS-AFM movie of CA lattice at pH 8. Scale bar corresponds to 10 nm. Acquisition time is indicated on the right top corner of the movie. Yellow square delineates the hexamer shown in Movie 2.

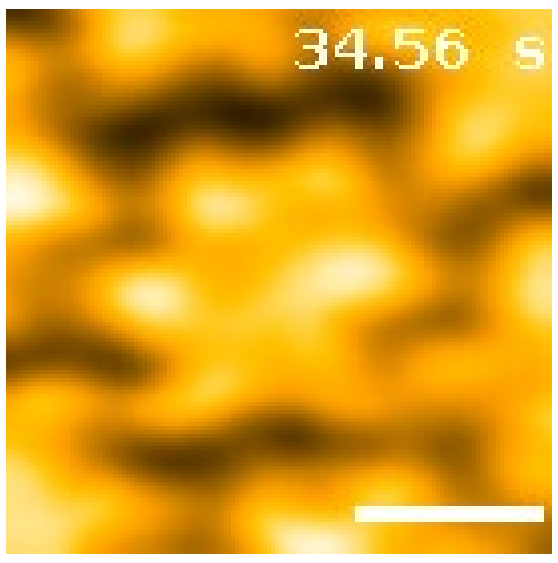

Movie S2. Frame extracted from Movie 2 showing single CA hexamer dynamics. Scale bar corresponds to 5 nm. Acquisition time is indicated on the right top corner of the movie.
